# Extensive Field Observations Throw Light on the Evolution of Mimicry in *Camponotus lateralis* (Hymenoptera: Formicidae)

**DOI:** 10.1002/ece3.72530

**Published:** 2025-12-02

**Authors:** Herbert C. Wagner, Felix Kraker, Gregor Bračko, Enrico Schifani

**Affiliations:** ^1^ Institute of Biology University of Graz Graz Austria; ^2^ Department of Biology, Biotechnical Faculty University of Ljubljana Ljubljana Slovenia; ^3^ Department of Chemistry, Life Sciences and Environmental Sustainability University of Parma Parma Italy

**Keywords:** *Camponotus kiesenwetteri*, *Colobopsis truncata*, *Crematogaster scutellaris* group, Mediterranean region, preadaptations, trail‐following behavior

## Abstract

The Mediterranean ant‐ant association between *Camponotus lateralis* and *Crematogaster scutellaris* has fascinated naturalists for long, with a focus on documenting the attraction of *Ca. lateralis* workers to *Cr. scutellaris* trails. Little attention, in contrast, has been put on similar behaviors between other *Camponotus* and *Crematogaster* species. Moreover, neither the adaptive value of mimicry in *Ca. lateralis*, which mimics the color patterns of different *Crematogaster* species, nor its evolution is understood. Data and video records (https://figshare.com/s/b627084f6a7f60e6dc4c) of 2 one‐month‐long and several short field trips brought insights into this mimicry evolution: We recorded trail following, a behavior hitherto known from four Mediterranean species, from 10 Camponotini species. The percentage of *Ca. lateralis* workers to those of *Crematogaster* on trails was only 2.4%. *Camponotus lateralis* , *Ca. ruber*, and *Colobopsis* species were the only trail followers mimicking *Crematogaster* color patterns. Dealate gynes of *Ca. lateralis* and *Ca. kiesenwetteri* founded colonies next to *Crematogaster* trails. Of 211 *Ca. lateralis* colonies, 79% were associated with *Crematogaster*. *Camponotus lateralis* nests putatively associated with *Crematogaster* had 2.2 times more workers and a 2.2 times higher chance to produce sexuals. *Camponotus lateralis* major workers defended entrances against *Crematogaster* by blocking them with their heads, in a similar manner as *Colobopsis*. At 15 of 19 sites, lacertid lizards were present; some ingested ants but avoided *Crematogaster*. Several characteristics of *Ca. lateralis* concerning its behavior, morphology, colony structure, and nesting site selection are advantageous for the mimicry strategy. Of these, we identified those shared with related non‐mimetic species as putative preadaptations. The manifestation of regional color morphs could be the only species‐specific mimicry adaptation, while several common characteristics among Mediterranean Camponotini appear as preadaptations to trail following or to mimicry. Trail following facilitates access to food and protection; color mimics likely evolved from non‐mimetic trail followers and enjoy reduced predation through Batesian mimicry.

## Graphical abstract

*Camponotus lateralis* (C, D) mimics regional color morphs of *Crematogaster* (A, B). Field trips brought insights into ecology, trail sharing, sexual reproduction, colony foundation, nest defense, and predation pressure of this mimetic ant. We found that the mimicry is Batesian and suggest several preadaptations for its evolution.

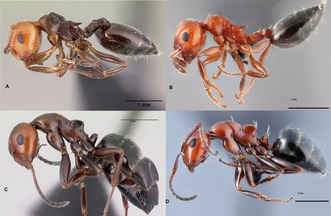

## 1 Introduction

The association of the formicine ant *Camponotus lateralis* (Olivier, 1792) with the myrmicine ant *Crematogaster scutellaris* (Olivier, 1792), two Mediterranean species, has fascinated naturalists for more than a century (Gené 1842; Emery 1886, 1915; Forel 1898; Müller 1923). Trail‐following behavior is a key characteristic of this association: Workers of *Ca. lateralis*, a subdominant and nimble species, often occur near species of the *Cr. scutellaris* group (*Cr. scutellaris*, *Cr. schmidti* (Mayr, 1853), *Cr. ionia* Forel, 1911) and follow their trails. These *Crematogaster* species form huge colonies, forage through long permanent trails, possess an effective repellent venom, and are territorial and aggressive (Seifert 2018; Video 1 in Supplement). When *Ca. lateralis* workers meet those of *Crematogaster*, they react quickly and sidestep to avoid aggression before returning to following their trails (Goetsch 1942, 1951, 1953; Kaudewitz 1955; Baroni Urbani 1969; Carpintero et al. 2005; Menzel, Woywod, et al. 2010). *Camponotus lateralis* can follow *Cr. scutellaris* to aphid colonies, which may suggest that this behavior improves its ability to locate food resources (Goetsch 1942, Goetsch 1951; Baroni Urbani 1969; Carpintero et al. 2005; Video 2 in Supplement). The usage of *Crematogaster* trails has been observed in other species of *Camponotus* all over the world as well as in another camponotine ant genus, *Colobopsis* (Davidson 1988; Ito et al. 2004; Vantaux et al. 2007; Menzel, Linsenmair, and Blüthgen 2008; Menzel et al. 2014; Menzel, Pokorny, et al. 2010; Schifani et al. 2022; Pérez‐Delgado and Wagner 2024; Table A1). While more efforts have been put into investigating the behavior of *Ca. lateralis* in trails of *Cr. scutellaris*, overall lesser attention has been given to this behavior in other Mediterranean *Camponotus*, *Colobopsis*, and *Crematogaster* species (but see Forel 1898; Goetsch 1942, Goetsch 1951; Stukalyuk and Radchenko 2011; Wagner 2019; Schifani et al. 2022; Stukalyuk et al. 2023).

**FIGURE 1:**
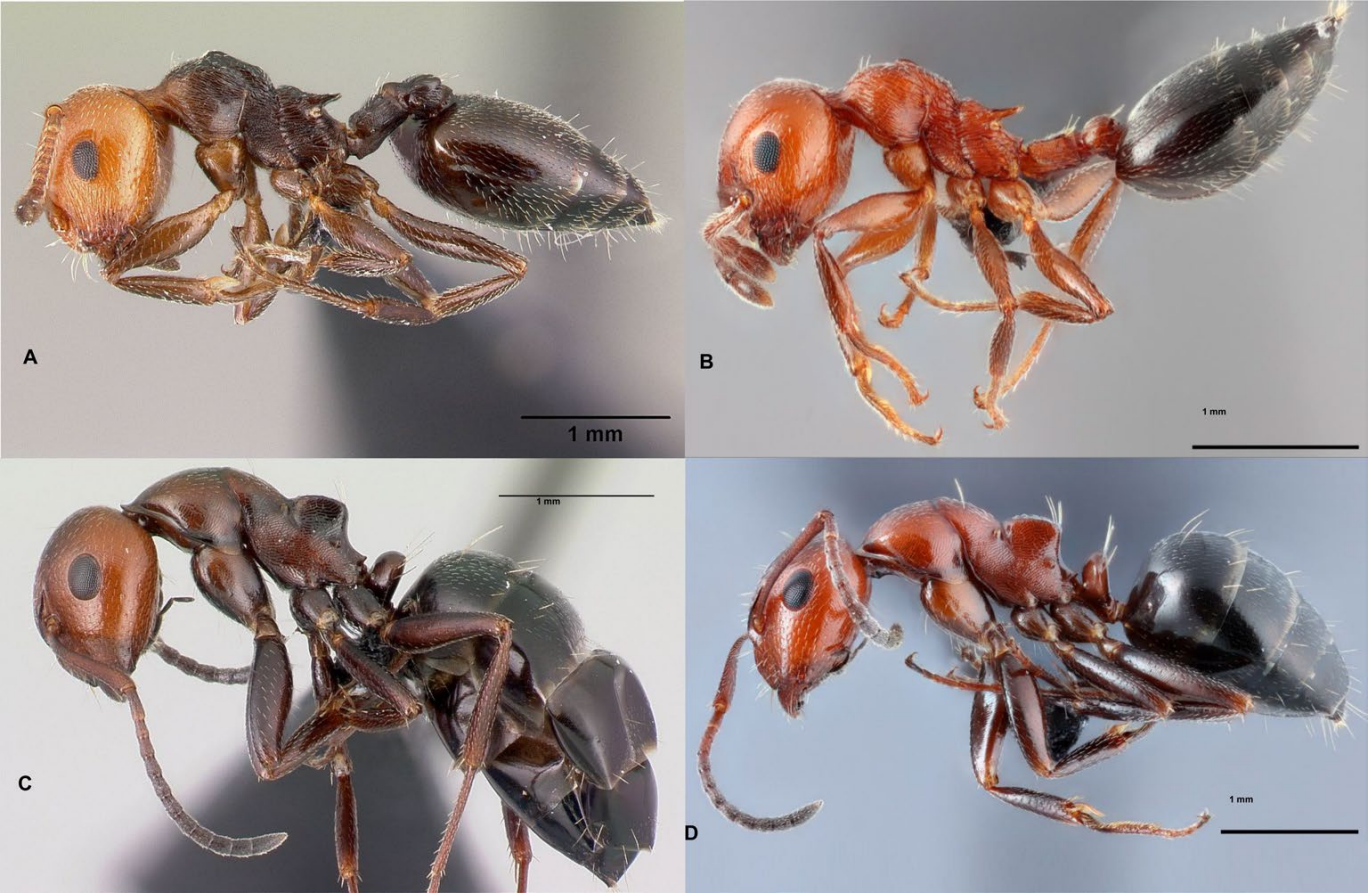
Comparison of two model species (A: *Crematogaster scutellaris* ; B: *Cr. schmidti*) with two color morphs of the mimetic species *Ca. lateralis* (C: *scutellaris*‐like color morph; D: *schmidti*‐like color morph). Pictures of the models (A, B) are placed above their mimics (C, D). Photos: A: E. Prado (casent0179890, AntWeb); B: G. Bračko; C: E. Prado (casent0179871, AntWeb); D: G. Bračko.

The second key characteristic of the association between *Ca. lateralis* and the *Cr. scutellaris* group is visual mimicry, a feature that often coexists with trail following in ant‐ant associations (Ito et al. 2004; Gallego Ropero and Feitosa 2014; Schifani et al. 2022; Pérez‐Delgado and Wagner 2024; Table A1). Frequently, *Ca. lateralis* shares the color pattern, a reddish head and a dark brown to blackish rest of the body, with *Cr. scutellaris*, which most authors interpreted as a clear case of color mimicry (Emery 1886; Goetsch 1942, 1951, 1953; Kaudewitz 1955; Bernard 1967; Menzel, Woywod, et al. 2010; Wagner 2013, 2014; Seifert 2019; Schifani et al. 2022). In sympatry with *Cr. schmidti*, *Ca. lateralis* frequently also exhibits a reddish mesosoma (Figure 1; Wagner 2014; Seifert 2018, 2019). It is unclear if, in addition to color, size also plays any role in this mimicry (cf. Kaudewitz 1955). At least one other ant species of the region, *Colobopsis imitans* Schifani et al. 2022, offers an example of convergent evolution with *Ca. lateralis*, exhibiting the same mimicry color pattern and the same trail‐following behavior of *Cr*. *scutellaris*. Perhaps a looser association involves *Colobopsis truncata* (Spinola, 1808) as a mimic of *Dolichoderus quadripunctatus* (Linnaeus, 1771) (Schifani et al. 2022). Clear or suspected cases of mimicry of *Cr. scutellaris* are also observed in *Camponotus ruber* Emery, 1925, in some other insects, including mirid bugs and braconid wasps, and in spiders (Schifani et al. 2022, 2024). A similar system has recently been uncovered on the Canary Islands, where *Ca. guanchus* Santschi, 1908 exhibits trail following and color mimicry of a local *Crematogaster* species, *Cr. alluaudi* Emery, 1893, which is also mimicked by at least two hemipteran species (Pérez‐Delgado and Wagner 2024).

Four hypotheses on the adaptive value of mimicry in these ants have been proposed, not necessarily excluding one another. However, none of them has gathered conclusive experimental evidence:

- Batesian mimicry: Emery (1886) suggested a bad taste of *Crematogaster* and an anti‐predatory adaptation in *Ca. lateralis*, being the first to suggest a Batesian‐mimicry mechanism. Marlier et al. (2004) suggested the colors of *Cr. scutellaris* to be aposematic, a chemical defense of *Cr. scutellaris* against vertebrate predators, and Batesian mimicry of *Ca. lateralis*. Menzel, Woywod, et al. (2010) mentioned birds and spiders as putative predators in this system. Lizards too have been proposed, as the Italian wall‐lizard *Podarcis siculus* (Rafinesque‐Schmaltz, 1810) was observed ingesting workers of the *Ca. lateralis* group but avoiding those of *Cr. scutellaris* under laboratory conditions (Wagner 2013, 2014). Schifani et al. (2022) noted how *Cr. scutellaris* workers are much more aggressive and better equipped with chemical defenses than their mimics *Ca. lateralis* and *Co. imitans*, and believed Batesian mimicry to be the most likely hypothesis. In a recent study (Wagner 2025), remains of *Cr. scutellaris* and *Ca. lateralis* were assessed to be strongly underrepresented in feces of ant‐eating *Podarcis muralis maculiventris* (Werner, 1891) lizards; the uneven consumption in relation to trophic availability was interpreted as a consequence of bad taste and aposematism in *Cr. scutellaris* and Batesian mimicry and trail sharing in *Ca. lateralis*.
- Müllerian mimicry: This hypothesis would apply to theoretical predators that do not have any preference for *Cr. scutellaris* or its mimics but dislike them both equally (Schifani et al. 2022). Both *Ca. lateralis* and *Co. imitans* can release some formic acid in self‐defense, which for some predators could potentially be similarly repelling as the aggressiveness and chemical defense of *Cr. scutellaris*.
- Dilution effect: Schifani et al. (2022) speculated that, for both *Ca. lateralis* and *Co. imitans*, mimicry of *Cr. scutellaris*, combined with close spatial proximity due to trail following and the much greater numeric abundance of the latter (whose colonies have a much larger population size (Stukalyuk et al. 2022)) would result in a dilution effect (Lehtonen and Jaatinen 2016): Hypothetical predators with no preference for *Cr. scutellaris* or its mimics would be unlikely to prey upon the much rarer mimic workers on the trails, potentially even more so due to the greater speed and agility of *Ca. lateralis* and *Co. imitans*.
- Avoiding attacks by *Crematogaster* ants: In contrast to the hypotheses i–iii, Goetsch (1942) considered the model *Cr. scutellaris* itself to be a selective force towards color mimicry. He described how *Cr. scutellaris* workers evaluated the color of *Ca. lateralis*, and that similarity to their own colony members reduced their aggression. Kaudewitz (1955) reported that the few *Ca. lateralis* workers with dark heads were significantly more often attacked than the mimicking ones. If this hypothesis proves to be true, it would be described as a case of aggressive mimicry (Wickler 1968) to exploit food resources of *Crematogaster*.

This study aims not only to evaluate the four mimicry hypotheses but mainly to address questions regarding the evolution of mimicry by comparing characteristics of *Ca. lateralis* with related species and to identify potential preadaptations and adaptations to mimicry. Menzel et al. (2014) suggested that some preadaptations could have favored the evolution of associations between *Camponotus* and *Crematogaster*. Focusing on chemical‐ecology aspects, they compared two tropical *Camponotus–Crematogaster* species pairs as well as *Ca. lateralis* and *Cr. scutellaris* and pointed out that *Camponotus* can perceive *Crematogaster* trail‐pheromones and that there are parallel shifts in cuticular‐hydrocarbon profiles between *Camponotus* and *Crematogaster*. However, in these cases, two of three *Camponotus–Crematogaster* pairs were bound by a mutualistic association of nest‐sharing (parabiosis), suggesting rather different evolutionary pressures than what is observed in the Mediterranean.

We addressed eight categories of research questions:

### 1.1 Nest Sites, Densities, and Population Sizes

Where are the nests of *Camponotus lateralis* ? How large are nest densities and nest populations? Small nest densities and populations in relation to *Crematogaster* would be in line with hypotheses i (Batesian mimicry) and iii (dilution effect), but not with hypothesis ii (Müllerian mimicry). Colony sizes smaller than in related species would mean that this trait was a specific adaptation to mimicry, while no difference from related species would suggest small colony sizes to be a preadaptation.

### 1.2 Trail‐Following Behavior

Observations of trail‐following behavior of *Ca. lateralis* and further camponotine ants should give hints of evolutionary pathways towards mimicry. In detail, a wide spread of trail‐following species among camponotine ants, including also non‐mimics phylogenetically related to mimics, would suggest that trail following was first and mimics evolved out of trail followers. Vice versa, a wide spread of mimicry among camponotine ants, including also non‐trail‐following species phylogenetically related to trail followers, would suggest that mimicry was first and trail followers evolved out of mimics. Which species perform trail following at which frequency, and how large is the portion of trail followers in comparison with *Crematogaster*? Are minor workers, which match in size with those of *Crematogaster*, overrepresented in comparison with the larger major workers? These questions seek insight into the evolution of the association between the two genera. Moreover, the portion of trail followers versus models can provide hints regarding hypotheses i, ii, and iii, that is, Batesian mimicry, Müllerian mimicry, and the dilution effect. Batesian mimics are numerically less frequent than their models, an argument which also holds true for the dilution effect (Wickler 1968).

### 1.3 No Color‐Based Aggression in *Crematogaster*

Species of the *Crematogaster scutellaris* group show distinct differences in coloration (Figure 1); *Cr. scutellaris* is usually blackish with a reddish head. Goetsch's (1942) and Kaudewitz' (1955) hypothesis of nestmate discrimination via color in *Cr. scutellaris* made it necessary to put special attention on the aggression of *Crematogaster* workers against camponotine trail‐followers as well as against nestmates with abnormal color. Is *Crematogaster* more aggressive against camponotine trail‐followers with deviating than to those with congruent color? Is *Crematogaster* aggressive against nestmates deviating in color from normal ones? Answering these questions aims to test hypothesis iv, the hypothesis of aggressive mimicry.

### 1.4 Putative Associations With *Crematogaster*

How frequent is the association between *Ca. lateralis* and related species with *Crematogaster*? Do all *Crematogaster* species of the *scutellaris* group in the region have the same suitability for the association? Is there a relation between population size or sexual production and the association with *Crematogaster*? To test the fitness success of nests with and without such an association, worker numbers and sexual production were compared. A distinct fitness advantage of *Ca. lateralis* nests associated with *Crematogaster* would indicate a close ecological relationship—possibly even a dependency on *Crematogaster*.

### 1.5 Association With *Crematogaster* in *Camponotus* and *Colobopsis* Gynes

Do gynes of trail‐following Camponotine ants establish colonies in proximity to *Crematogaster* (as suggested by Baroni Urbani 1969; Menzel, Woywod, et al. 2010)? A positive answer to this question would underscore the importance of *Crematogaster* for trail followers, leading to the evolution of strategies that increase the chance of occurring in the *Crematogaster* territory. A wide spread of this strategy among Camponotine ants would suggest that it is a preadaptation to mimicry.

### 1.6 Defending Nest Entrances Against *Crematogaster*

It is unknown how small colonies with a few 100 submissive *Ca. lateralis* workers are able to survive close to *Crematogaster* colonies with a few 1000 to more than 100,000 (Stukalyuk et al. 2022 and citations herein) aggressive workers. Are there any strategies to avoid invasions of *Crematogaster* workers into the *Ca. lateralis* nests?

### 1.7 Mimicry

Regional color mimicry in *Ca. lateralis* has been suggested without quantitative proof (Wagner 2014; Seifert 2018, 2019; Figure 1). The existence of this phenomenon at least to some extent is obvious and quantitative details are currently investigated via color red‐green‐blue measurement (Kraker and Wagner 2025). Here, we focus only on a few newly detected color‐mimicry aspects that can be described qualitatively. Does *Ca. lateralis* also mimic the dark brown to blackish color of *Crematogaster* on Crete? Which additional camponotine trail‐followers mimic the (regional) color patterns of *Crematogaster* models? These questions aim to understand the frequency and (convergent) evolution of mimicry in Mediterranean camponotine ants, as well as its relation to trail‐following behavior (see Section 1.2 | Trail‐following behavior).

### 1.8 Field Observations on Predators

Which predators come into question to drive the evolution of color mimicry in *Ca. lateralis*? Is Batesian (hypothesis i) or Müllerian (hypothesis ii) mimicry more likely based on animals interacting with *Ca. lateralis*?

## 2 Materials and Methods

**FIGURE 2:**
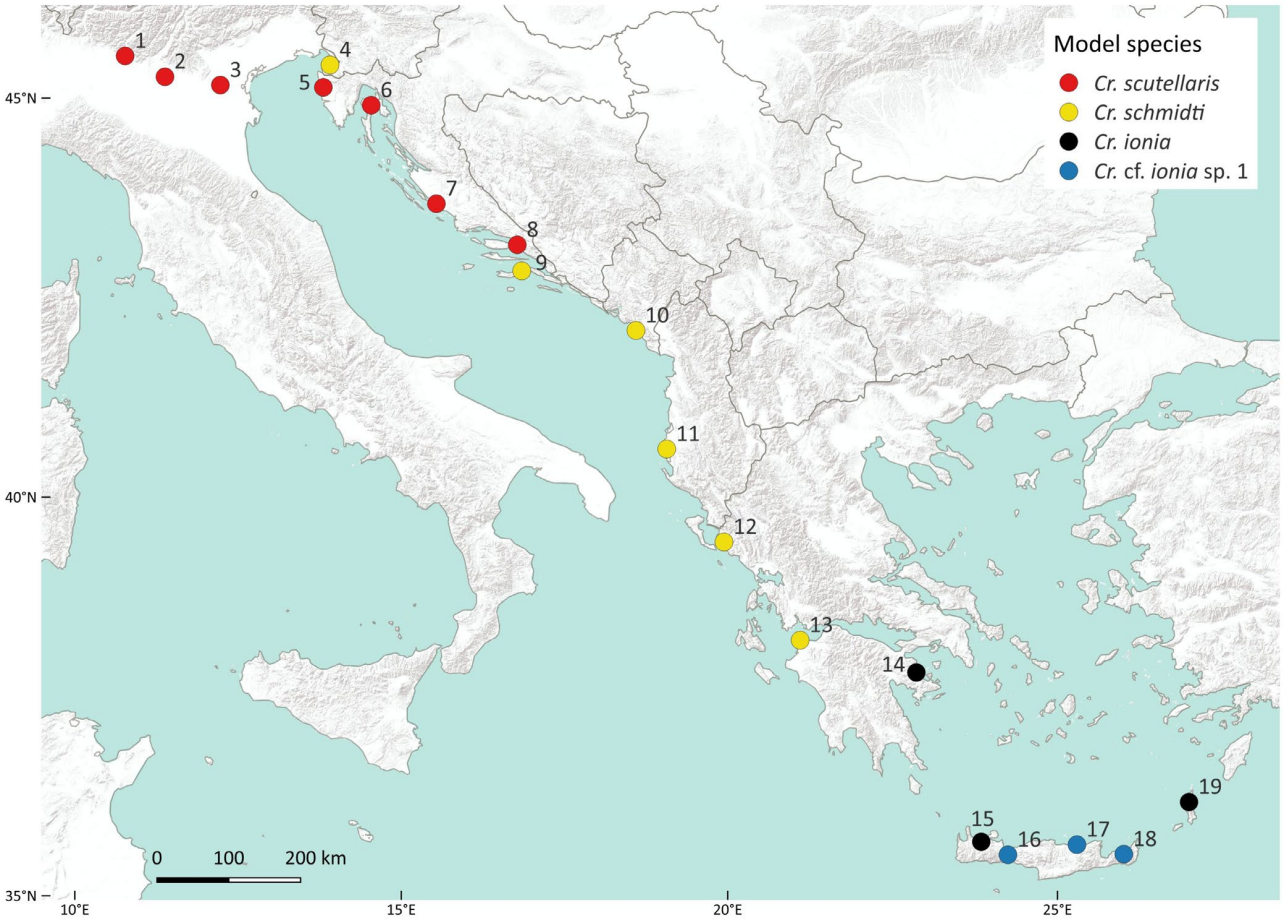
Nineteen main sites for field observations and sampling in this study; *Crematogaster* model species are shown for each site.

Observations were made systematically at 19 main sites from Iseo Lake (Italy) in the northwest to the island Karpathos (Greece) in the southeast of the studied area (Table A2, Figure 2). In order to collect ants in Greece, a permit from the Ministry of Environment and Energy was obtained. Two main sites, 4 and 5, were divided into two nearby subsites each (4a and 4b as well as 5a and 5b), whereas subsites a and b each differed in their ecological conditions. Only near‐natural biotopes in which at least five *Ca. lateralis* nests were located were accepted as main sites, regardless of *Crematogaster* presence or absence. Sizes of ecologically homogenous biotopes were estimated in the field by pacing and afterwards controlled using the polygon tool in Google Earth Pro. 18 main sites were visited by HCW; main site 11 by FK. In dependence on nest density and structural diversity of each biotope, 7 to 15 h per main site were spent. Random observations from nine additional sites (Table A2) completed the data set. The behavior of ants was video‐recorded using a smartphone. The videos are available under https://figshare.com/s/b627084f6a7f60e6dc4c. Ant material was collected in pure ethanol for subsequent identification, which was carried out following the keys of Seifert (2018, 2019). For statistics, Fisher's exact tests (R. A. Fisher 1922, 1925) via the online tool https://www.socscistatistics.com/tests/fisher/default2.aspx (Stangroom 2024) and 2‐sided *t*‐tests (Student 1908) were performed. An *α*‐level of 0.05 was used.

### 2.1 Nest Sites, Densities, and Population Sizes

*Camponotus lateralis* nests were searched for by following foragers to their nests, breaking dead wood, or turning over dead wood or stones. Nests of other species of the *Ca. lateralis* group were not specifically searched for, but coincidental findings were recorded. Nest densities per area unit were estimated by applying a quick search *sensu* Seifert (2017). To get an idea of the nest population, nests were opened, and workers were roughly counted from the nest. To this number we added the number of workers that were foraging outside the nest. We estimated that their number was between 0% and 25% (cf. Seifert 2018 for the *Formica rufa* group and *Lasius fuliginosus* ) of the total nest population, depending on the weather conditions.

### 2.2 Trail‐Following Behavior

At each *Camponotus* and *Colobopsis* finding in association with *Crematogaster*, the numbers of workers of the trail‐following species and those of *Crematogaster* were counted for direct numerical comparison. In terms of space, the whole trail, which was accessible unhindered for the observer in the field from the ground level, typically a more or less vertical length of 2–3 m along a tree trunk, was considered. In terms of time, all ants simultaneously present along the observed trails were considered. Separately, the number of minor and major workers along the trails was counted over a 5‐min time span to calculate their ratio.

### 2.3 No Color‐Based Aggression in *Crematogaster*

We aimed to quantify the aggressiveness of *Crematogaster* against different camponotine trail‐followers as well as against nestmates with abnormal color. Of 101 videos analyzed, 88 were recorded at 17 of the 19 main sites (1–9, 11–16, 18, 19) and 13 on additional sites (a1, a6, a7, a9). Behavior was exclusively evaluated from video records on a 60 cm screen. We interpreted acceleration, direction change towards the trail follower, or opening the mandibles as aggression. If the trail follower sidestepped too fast for giving the *Crematogaster* worker the chance to show aggression, it was scored as non‐aggressive. Only encounters in which *Camponotus* workers frontally met with *Crematogaster* workers were considered. *Crematogaster* workers carrying items were excluded.

To quantify the aggressiveness against nestmates, we studied a single *Cr. scutellaris* colony (main site 5b) where we observed four workers with fully blackish heads. A trail of the colony was video‐recorded to detect putative aggression of normal workers against those with blackish heads.

### 2.4 Putative Associations With *Crematogaster*

Since trail following could not always be observed (e.g., if weather does not allow ants to forage or if *Camponotus* colonies were very small), a category of putative associations was created. We defined a *Camponotus* nest to be putatively associated with *Crematogaster*, if at least one of the following conditions was met:

1. Workers were observed in a *Crematogaster* trail.
2. *Crematogaster* workers were observed in a distance of less than two meters to the nest of *Camponotus*.
3. The minimal distance between centers of the nest of *Camponotus* and those of *Crematogaster* is less than 5 m.

All trail‐following *Camponotus* species were considered.

For the evaluation of the presence or absence of alates (winged gynes and males) in *Ca. lateralis* nests, only nests which could be opened mechanically and collected in March, April, or October (but not from May to August) were considered, because sexuals are produced in summer, hibernate in the nest, and swarm mostly in May (Seifert 2018; Borovsky et al. 2022). Only presence or absence was evaluated while a single alate was enough for a positive count. Due to limited time in the field, counting alates would have induced considerable error, as individuals in nest chambers within solid wood—unopenable by mechanical means—were likely to be overlooked. Estimating the numbers was avoided in order to minimize the risk of unintentionally biasing the results towards the hypothesis.

### 2.5 Association With *Crematogaster* in *Camponotus* and *Colobopsis* Gynes

In parallel with scanning the surface of different substrates (trees, soil, etc.) to detect workers at main sites 1–19, attention was also given to dealate gynes, which are easier to detect due to their larger size. The number of gynes was counted taking note of how many of them were in proximity to *Crematogaster* nests or trails (i.e., at a distance less than 100 cm). Behavioral interactions between *Camponotus* gynes and *Crematogaster* workers were video‐recorded. We defined antennation as a fast movement of the antennae towards a specific object. Typically, the gyne is moving slowly and often changing direction during antennation.

### 2.6 Defending Nest Entrances Against *Crematogaster*

A nest entrance of *Ca. lateralis* (main site 13), well accessible for observations and close to a *Cr. schmidti* trail, was observed for 20 min. About 100 *Cr. schmidti* workers were taken from a nest nearby; since these workers were defending their nest against the collector, they were aggressive (running on hands and biting). They were put in front of the *Ca. lateralis* nest entrance to observe the nest defending strategy of *Ca. lateralis* and the putative aggression of *Crematogaster*. In *Crematogaster*, the behavioral categories “avoiding the entrance” (i.e., returning or changing direction after reaching the nest entrance by chance), “biting *Ca. lateralis*”, “besmearing *Ca. lateralis* with venom”, and “walking into the nest” were analyzed.

### 2.7 Mimicry

Color patterns of camponotine trail‐followers were compared with those of the associated *Crematogaster* species. New and obvious cases are presented here via verbal descriptions, pictures, and videos.

### 2.8 Field Observations on Predators

Each main site was investigated for the presence of lizard predators. All lizards occurring syntopically with *Ca. lateralis*, that is, occurring at the same site and in the same biotope type, were considered. They were determined using Kwet (2022). Feces or regurgitated material was collected and any ant material in it identified. Densities were estimated directly in the field by counting individuals per area and later verified by calculating biotope sizes using Google Earth Pro. Anecdotes concerning interactions between lizards and ants of the mimicry system, or phylogenetically related to *Ca. lateralis*, were observed and—when possible—video‐recorded. A video was also used to calculate the speed of an ant during the interaction with a lizard. In one case, an adult male and an adult female of *Podarcis erhardii* (Bedriaga, 1882) were fed with minor and major workers of *Ca. dalmaticus* (Nylander, 1849) to observe putative ingestion.

## 3 Results

### 3.1 Nest Sites, Densities, and Population Sizes

Of 142 located nests of *Ca. lateralis*, 134 (94%) were in different kinds of wood, in most cases in dead branches with 1–10 cm thickness lying on the ground (details in Table A3); mostly in *Quercus* or *Pinus* (details in Table A4). Only 6% of nests were in stone walls or in the soil (Video 3 in Supplement). Nest densities were 1.7 ± 1.4 (max = 4.5, *n* = 9) nests/100 m^2^ in *Quercus*‐dominated broadleaf forests, 1.3 ± 0.7 (max = 2.5, *n* = 7)/100 m^2^ in *Pinus* forests, and 2.4 ± 1.3 (max = 4, *n* = 4) in Cretan humid *Platanus*‐*Eucalyptus* forests (Table A5). We estimate that the mean nest population of *Ca. lateralis* was 105 ± 106 (min = 5, max = 700; *n* = 113) workers; the population size decreased with latitude (2‐sided *p* < 0.001, *R* = −0.3335; Table A5). At all sites, one species of the *Crematogaster scutellaris* group was present (Figure 2, Table A2).

*Camponotus piceus* (Leach, 1825) was found in more xerophilous and more open biotopes than *Ca. lateralis*, typically on wood margin or in bushland. *Camponotus dalmaticus* (*n* = 9 main sites) and *Ca. rebeccae* Forel, 1913 (*n* = 3 sites) were found on humid sites, always syntopically with *Ca. lateralis* and with similar or identical nesting preferences (Table A3). The only obvious ecological difference to *Ca. lateralis* was the statistically different probability of being associated with *Crematogaster* (see Section 3.4 | Putative associations to *Crematogaster*).

### 3.2 Trail‐Following Behavior

Following trails of the four species of the *Crematogaster scutellaris* group, *Cr. scutellaris*, *Cr. schmidti*, *Cr. ionia*, and *Cr. ionia* cf. *ionia* sp. 1 *sensu* Salata et al. (2020) were directly observed in *Camponotus lateralis* (69 nests), *Ca. kiesenwetteri* (16), *Ca. dalmaticus* (7), *Colobopsis truncata* (7), *Co*. cf. *truncata* (7; these are samples from Crete and the Peloponnese), *Ca. gestroi* Emery, 1878 (4), *Ca. piceus* (1), *Ca. rebeccae* (1), *Ca. ruber* (1), and *Ca. spissinodis* Forel, 1909 (1) (Videos 4–19 in Supplement, Table 1). The majority of these species are not mimics (see Section 3.7 | Mimicry). In addition to ants, four individuals of a myrmecophilous silverfish (Zygentoma: Nicoletiidae) were also observed following trails of *Cr. ionia* (Video 20 in Supplement) and one of *Cr. schmidti*.

**TABLE 1:** Nest numbers of direct observations of trail‐following behavior.

Species	*Cr. scutellaris*									*Cr. schmidti*							*Cr. ionia*					*Cr*. cf. *ionia* sp. 1			Total
Site	1	2	3	5	6	7	8	a1	a2	4	9	10	11	12	13	a6	14	15	19	a7	a9	16	17	18	
*Ca. dalmaticus*											1		1	1	3	1									7
*Ca. gestroi*															1		1				1	1			4
*Ca. kiesenwetteri*																	5		5		1		3	2	16
*Ca. lateralis*	2	2	4	1	3	1	1			3	1	2	3	2	5		6	8	7	1		6	4	7	69
*Ca. piceus*				1																					1
*Ca. rebeccae*																								1	1
*Ca. ruber*									1																1
*Ca. spissinodis*								1																	1
*Co. truncata*	1	1	1							1		3													7
*Co*. cf. *truncata*															1			3				2		1	7
Total	20									29							38					27			114

In *Ca. lateralis* nests associated with *Crematogaster*, the majority of the observed foragers used *Crematogaster* trails. The percentage of all *Ca. lateralis* workers on *Crematogaster* trails was 2.4% in relation to those of *Crematogaster* (*n* = 233 *Ca. lateralis* and 9607 *Crematogaster* workers of 75 (10 zero samples included) associated *Ca. lateralis* nests), in *Ca. kiesenwetteri* 1.9% (*n* = 56 *Ca. kiesenwetteri* and 2882 *Crematogaster* workers of 16 associated nests). Trail following was observed in *Ca. lateralis* (*n* = 69 of 211 colonies) significantly more often than in *Ca. dalmaticus* (*n* = 7 of 68) and *Ca. piceus* (*n* = 1 of 39; two‐sided *p* values each < 0.001), but not significantly different often from *Ca. gestroi* (4 of 14), *Ca. kiesenwetteri* (14 of 40), *Ca. rebeccae* (1 of 9), and *Colobopsis* (*n* = 14 of 28) (two‐sided *p* values = 1.000, 0.855, 0.278, and 0.091, respectively).

In *Ca. lateralis*, 9.8% of workers (*n* = 338 workers of 69 nests) following *Crematogaster* trails were majors; under the assumption that 24% of workers are majors (Seifert 2018), they were highly significantly underrepresented in *Crematogaster* trails (two‐sided *p* < 0.001). In *Ca. kiesenwetteri*, 11.2% of workers (*n* = 107 workers of 16 nests) with trail‐following behavior were majors. The portion of majors on *Crematogaster* trails between both *Camponotus* species was not significantly different (two‐sided *p* = 0.713).

### 3.3 No Color‐Based Aggression in *Crematogaster*

*Camponotus* and *Colobopsis* workers, no matter which species or whether minor or major, showed the same behavior when making contact with a *Crematogaster* worker (whether frontally with its head or with its gaster): They recoiled and/or sidestepped avoiding further interaction before quickly returning to the trail. In all cases, *Crematogaster* workers attacked accessible trail followers that did not escape quickly enough. Biting behavior against *Ca. lateralis* occurred three times (Video 21 in Supplement). *Crematogaster* workers showed aggression against *Ca. lateralis* and non‐mimetic *Camponotus* workers (*Ca. dalmaticus*, *Ca. rebeccae*, *Ca. piceus*, *Ca*. *spissinodis*, *Ca. gestroi*, and *Ca. kiesenwetteri*) in 180 and 97 encounters, respectively, but no aggression (because of recoiling and/or sidestepping) in 179 and 112 encounters (Table 2, Videos 4–19 in Supplement); the difference was not significant (two‐sided *p* = 0.434). Vice versa, there was no case in which any trail follower was aggressive against *Crematogaster*. If two *Camponotus* or *Colobopsis* workers of the same colony met each other on or near a *Crematogaster* trail (*n* = 42), in 15 cases at least one worker recoiled (Video 22 in Supplement), and in seven cases at least one worker accelerated and ran away from its own nestmate (Video 23 in Supplement). Of 350 workers of *Cr. scutellaris* observed in a trail of a colony at main site 5b, four were fully black‐headed and thus distinctly different from the normal red‐headed ones. In 81 direct encounters, no normal worker showed aggressive behavior against a black‐headed one (Video 24 in Supplement) (although none of them recoiled and/or sidestepped); the difference to *Ca. lateralis* as well as to non‐mimetic *Camponotus* trail‐followers was highly significant (two‐sided *p* values each < 0.001).

**TABLE 2:** Numbers of aggressive and non‐aggressive behavior in *Crematogaster* workers (the *n* corresponds to *Crematogaster* worker individuals) against trail followers of different species and against conspecific individuals of different colors when encountering on trails.

Trail follower	*Crematogaster* aggression	No *Crematogaster* aggression
*Ca. dalmaticus* (5 nests at 5 sites)	13	14
*Ca. gestroi* (3 nests at 3 sites)	11	12
*Ca. kiesenwetteri* (11 nests at 5 sites)	71	78
*Ca. lateralis* (29 nests at 18 sites)	180	179
*Ca. piceus* (1 nest)	0	6
*Ca. rebeccae* (1 nest)	0	2
*Ca. spissinodis* (1 nest)	2	0
*Co. truncata* (3 nests at 3 sites)	7	6
*Co*. cf. *truncata* (3 nests at 2 sites)	9	7
All *Camponotus* without *lateralis* (22 nests on 11 sites)	97	112
Black‐headed *Cr. scutellaris* (1 nest)	0	81

### 3.4 Putative Associations With *Crematogaster*

In *Camponotus lateralis* , 166 of 211 (79%) nests detected in near‐natural biotopes were putatively associated with *Crematogaster*. Vice versa, 149 of 200 *Crematogaster* nests (75%) were putatively associated with *Ca. lateralis*. In putative associations, the mean distance between nest centers was 284 ± 246 cm (10, 1400; *n* = 91). On sites with *Cr. scutellaris*, *Cr. schmidti*, *Cr. ionia*, and *Cr*. cf. *ionia* sp. 1, 50 of 72 (69%), 50 of 66 (76%), 36 of 38 (95%), and 29 of 34 (85%) *Ca. lateralis* nests were associated with *Crematogaster*, respectively. There were no significant differences between the four *Crematogaster* species (two‐sided *p* in all cases > 0.05). The portion of nests putatively associated with *Crematogaster* was very significantly lower in *Ca. dalmaticus*, *Ca. gestroi*, and *Ca. piceus*, in which 32 of 68 (47%), 6 of 14 (43%), and 9 of 39 (23%) were putatively associated with *Crematogaster* (two‐sided *p* < 0.001, 0.005, and < 0.001, respectively). In *Ca. rebeccae* and *Ca. kiesenwetteri*, no significant differences have been detected with seven of nine (78%) and 33 of 40 (83%) associated nests (two‐sided *p* values = 1.000 and 0.675), respectively.

Mean worker populations of putatively associated and putatively not associated *Ca. lateralis* nests were estimated to be 118 ± 112 (*n* = 89) and 53 ± 53 (*n* = 24), respectively; the former were very significantly higher than the latter by about 2.2 times (two‐sided *p* = 0.006).

Of *Ca. lateralis*, 130 nests could be opened mechanically and, consequently, the presence of alates could be evaluated. Of the 97 nests associated with *Crematogaster*, 51 (53%) contained alates. Of the 33 nests not associated with *Crematogaster*, 8 (24%) contained alates (Table 3). In other words: The chance of producing alates was 2.2 times higher in associated nests. The correlation was very significant (two‐sided *p* = 0.005).

**TABLE 3:** Presence of alates in *Camponotus lateralis* nests with and without *Crematogaster* association *Crematogaster*.

	With *Crematogaster*	Without *Crematogaster*	Sum
Alates	51	8	59
No alates	46	25	71
Sum	97	33	130

### 3.5 Association With *Crematogaster* in *Camponotus* and *Colobopsis* Gynes

Five dealate gynes of *Camponotus lateralis* at three sites (1 with *Cr. schmidti*, 2 with *Cr. ionia*, and 2 with *Crematogaster* cf. *ionia* sp. 1, at main sites 13, 15, and 16, respectively) and two of *Ca. kiesenwetteri* at one site (main site 16 with *Cr*. cf. *ionia* sp. 1) were associated with *Crematogaster*: They followed its trails, constantly antennating. They escaped from attacks of *Crematogaster* workers and visited wood openings in the surroundings of the *Crematogaster* trails, in one case only a few centimeters from the *Crematogaster* nest away (Video 25 in Supplement), in other cases ca. 30 cm next to the *Crematogaster* trail (Video 9 in Supplement). One dealate gyne of *Ca. lateralis* was detected in a founding chamber not in proximity to *Crematogaster*.

A gyne of *Colobopsis truncata* followed a trail of *Dolichoderus quadripunctatus* (a4) and was attacked by a worker (Video 26 in Supplement).

### 3.6 Defending Nest Entrances Against *Crematogaster*

**FIGURE 3:**
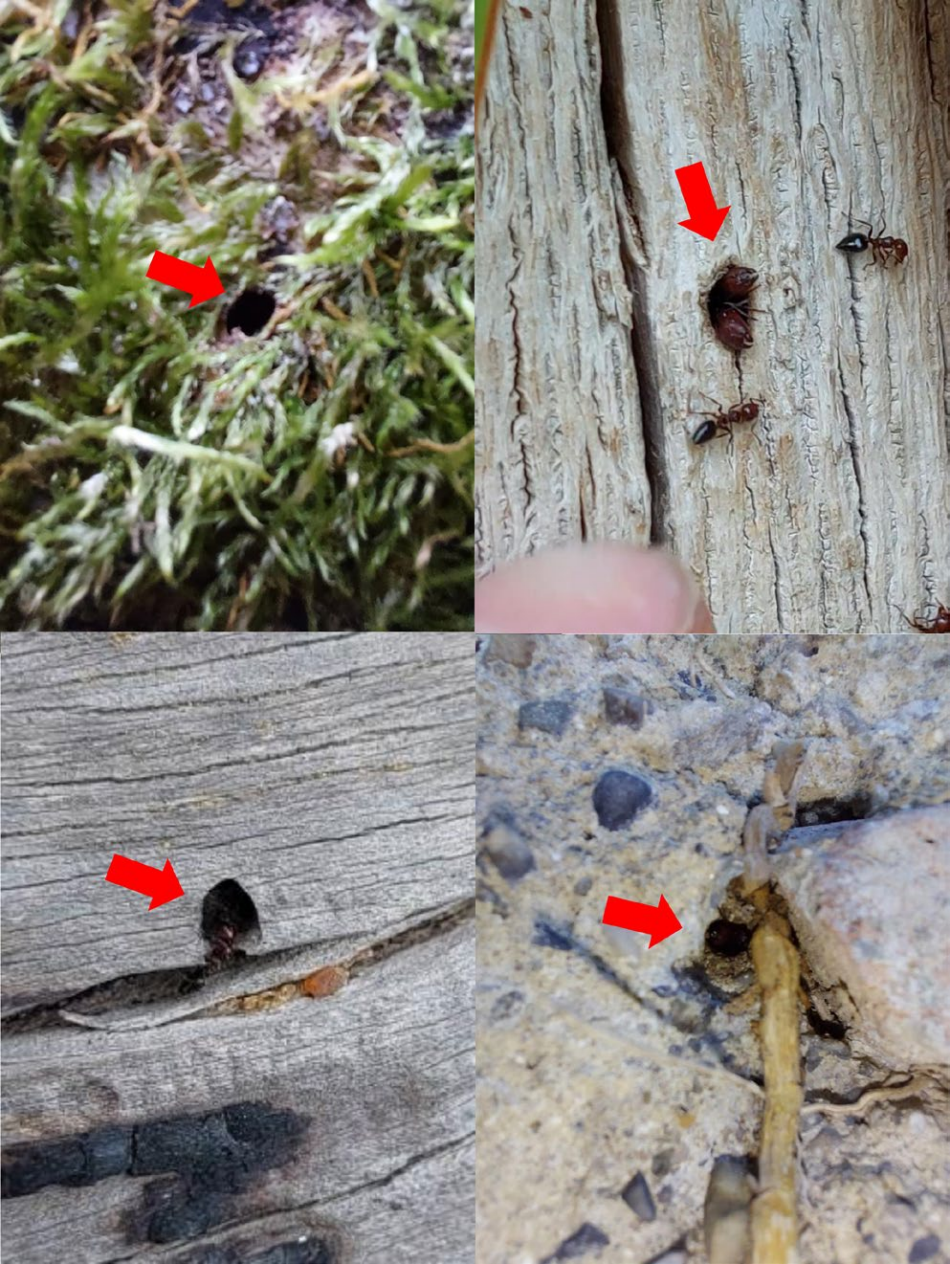
Four examples of nest entrances of *Camponotus lateralis* . Beginning from top left: At the base of a living *Ulmus* tree with moss (main site 4), at the base of a living *Quercus* tree (13), on a *Pinus* log on the ground (19), and in a wall crevice (1).

Entrances of 15 nests of *Camponotus lateralis* were detected. Two nests had two entrances, 13 had one—usually small—entrance (Figure 3). At a nest at main site 13, we observed the defending strategy against *Cr. schmidti*. About 100 *Crematogaster* workers were put close to the *Ca. lateralis* nest entrance of which 32 found the way to the entrance; 24 of them avoided the entrance, 3 bit the *Ca. lateralis* majors, 3 besmeared the *Ca. lateralis* majors with the venom and escaped afterwards, and 2 pushed past the major workers and went into the nest. One to three *Ca. lateralis* major workers remained passive in their position and blocked the entrance with their heads without biting; they sidestepped only when a nestmate went in (Video 27 in Supplement). After both majors at the entrance were collected with the aspirator, further workers, mostly majors, from inside the nest moved up to overtake the blocker position. After they were aspirated too, further workers from inside the nest moved up again. Altogether, 14 major and three minor workers were available to defend the nest entrance.

### 3.7 Mimicry

**FIGURE 4:**
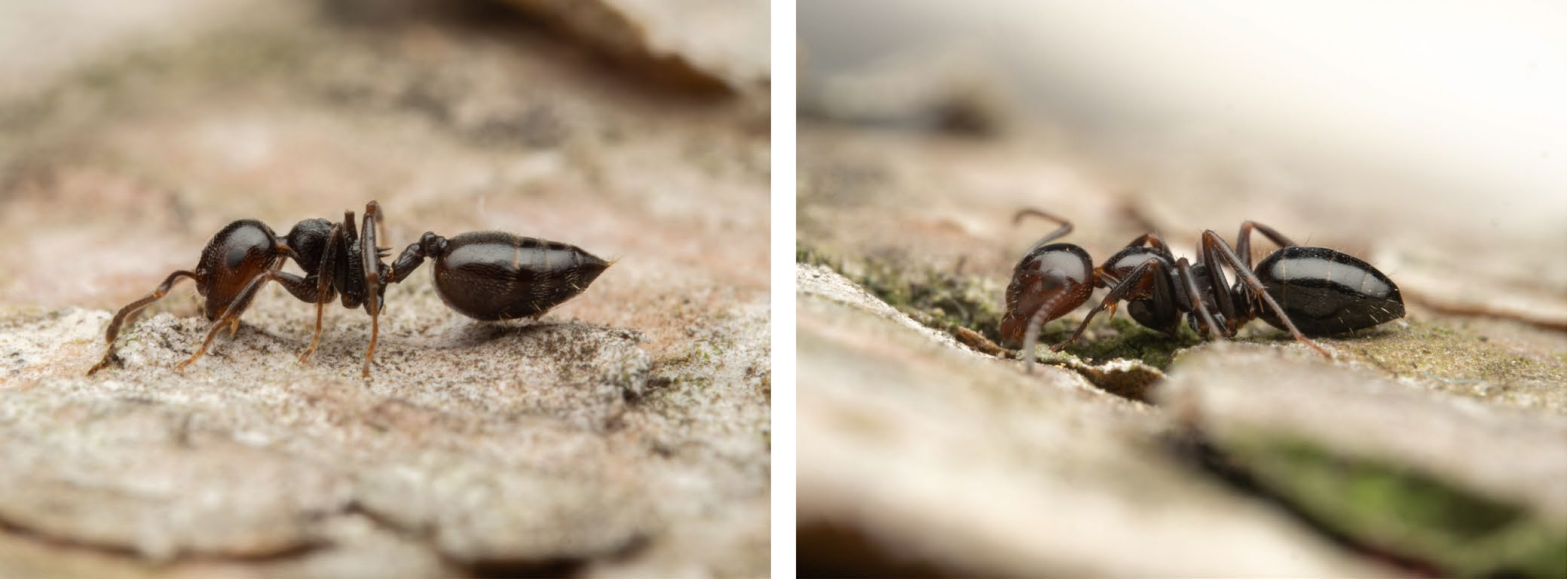
Left: Worker of *Crematogaster* cf. *ionia* sp. 1 from Crete with typical color (ID WAG3320). Right: Worker of *Camponotus lateralis* from Crete with typical color (ID WAG3333). The color pattern of this species resembles those of the sympatrically occurring species *Crematogaster ionia* and *Cr*. cf. *ionia* sp. 1, with which it is closely associated.

While the head color of almost all *Ca. lateralis* workers from sites 1–14 and 19 was more or less reddish, the heads of the workers from all nests at main sites 15–18 (Crete) were, with one exception (97%, *n* = 37), rather blackish with a slight brownish component on the genae, as in the sympatrically occurring model species *Cr. ionia* or *Cr*. cf. *ionia* sp. 1 (Video 7 in Supplement; Figure 4).

*Camponotus ruber* showed a pigmentation very similar to that of the sympatric *Cr. scutellaris* based on observations carried out in both Sicily and Tunisia (Video 19 in Supplement). The red coloration normally extends to most of the mesosoma, while the propodeum and the petiole may be blackish as the gaster. The overall proportion of red pigmentation is on average somewhat higher than in most *Ca. lateralis* of the same region.

At main sites 1–5, 10, and 13, all minor workers of *Colobopsis* had a reddish to brownish mesosoma (Videos 10 and 11 in Supplement). At sites 1–3, and 5, *Cr. scutellaris* occurred; at sites 4, 10, and 13, *Cr. schmidti* occurred. At sites 4, 5, and 10, *Dolichoderus quadripunctatus* was also found. At sites 15–18 and a8 (Crete), where *Cr. ionia* or *Cr*. cf. *ionia* sp. 1 but not *D. quadripunctatus* occurred, all 96 minor workers of ten *Colobopsis* cf. *truncata* nests had a fully dark brownish to blackish mesosoma (Video 12 in Supplement).

**FIGURE 5:**
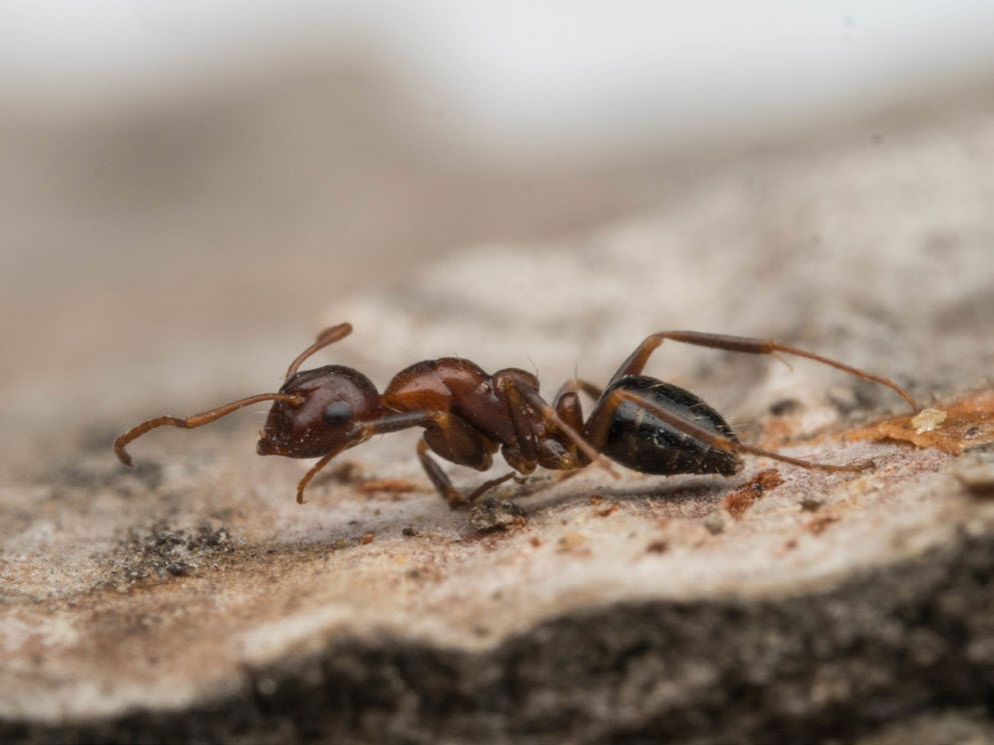
Worker of *Camponotus rebeccae* from Crete with typical color (ID WAG3352). The color pattern of this species differs from that of the sympatrically occurring species *Crematogaster ionia* and *Cr*. cf. *ionia* sp. 1, whose trails it rarely follows, by a reddish mesosoma.

*Camponotus kiesenwetteri* was always homogeneously gray and *Ca. gestroi* always blackish, no matter which *Crematogaster* species occurred syntopically. *Camponotus rebeccae*, of which workers of nine colonies were detected on main sites 16, 18, and a8, always had a reddish mesosoma (Figure 5), not mimicking the color of sympatric *Crematogaster* species (Figure 4).

To summarize, *Ca. lateralis*, *Ca. ruber*, *Co. truncata*, and *Co*. cf. *truncata* are mimics, while *Ca. dalmaticus*, *Ca. gestroi*, *Ca. kiesenwetteri*, *Ca. piceus*, *Ca. rebeccae*, and *Ca. spissinodis* are not mimics of *Crematogaster*.

### 3.8 Field‐Observations on Predators

True lizards (Lacertidae) as putative predators were observed at 15 of 19 main sites syntopically with *Ca. lateralis*, with maximum densities of 2–3 lizards/100 m^2^ at main site 4b (Table A6). At 11 localities, lizards were at the same tree or basal to the same tree as *Ca. lateralis*.

Seven anecdotes about predation behavior on ants by lizards and one by a gecko, are given in the following:

1. Main site 4b: One juvenile male of the Italian wall‐lizard (*Podarcis siculus*) was caught and spat out a large caterpillar, two ichneumonid wasps, and nearly all parts of a *Solenopsis* cf. *fugax* gyne. No material of *Crematogaster schmidti* , a very frequent species on the site, was among the regurgitated insect material.
2. Site 5b: A one‐year‐old male of *P. siculus* was ca. three meters away of two large nests of *Cr. scutellaris* and *Ca. lateralis*. It followed a single *Crematogaster* worker on a *Pinus* log lying on the ground and touched the worker with its tongue, after which it kept moving forward without further interacting with the ant.
3. Site 12: One adult female of the blue‐throated keeled lizard (*Algyroides nigropunctatus*), stood still near a trail of *Cr. schmidti* on an *Olea europaea* tree, and ignored seven workers of *Cr. schmidti* in front of its head. The workers touched the lizard with their antennae and circumvented it, except for one worker that walked over the lizard's tail (Video 28, 00:08:27, in Supplement). In contrast, a worker of *Cataglyphis* and one of *Ca. dalmaticus*, after touching the motionless lizard with the antennae, changed their course away from it and accelerated their speed (Video 28, 00:04:53 and 00:09:59, in Supplement). The latter had an initial speed of 0.04 m/s when it came down from the *Olea* tree with a full gaster. After touching the lizard first at a toe, it accelerated to 0.05 m/s. After it touched the lizard 0.6 s later on its stomach, it accelerated to 0.09 m/s and fell or jumped off the tree 0.8 s after the second touch. Feces of the same lizard individual (Video 28, 00:11:42, in Supplement) were examined and contained near all cuticula parts of an alate gyne of *Ca. dalmaticus*, of 1–2 beetles, and a spider. A male lizard of the same species nearby was also observed ignoring all *Cr. schmidti* workers it encountered while walking along their trail (Video 29 in Supplement).
4. Site 14: Two individuals of the Erhard's wall‐lizard (*Podarcis erhardii*), an adult male and an adult female, being close to nests of *Cr. ionia*, *Ca. lateralis*, *Ca. dalmaticus*, and *Ca. kiesenwetteri*, ingested each one major worker of *Ca. dalmaticus* thrown in front of them while they ignored minor workers (Video 30 in Supplement).
5. Site 16: We observed workers of *Ca. lateralis* and *Cr*. cf. *ionia* sp. 1 walking over a stone, which was the hiding place of an adult male of *Lacerta trilineata*, and on trees next to it. The lizard was observed ingesting other unidentified prey (larger than ants) but ignoring ant workers which walked over it (Video 31 in Supplement). Feces of this lizard contained one cockroach (Ectobiidae) and one or two spiders, but no ants.
6. At site a3, *P. siculus* lizards were regularly observed ignoring workers of *Cr. scutellaris* from trails. Over the course of several years, only once a lizard was observed preying upon multiple *Cr. scutellaris* workers from a trail.
7. At site a3, the moorish gecko *Tarentola mauritanica* (Linnaeus, 1758) has been observed to be a regular predator of *Cr. scutellaris* during nuptial flights, but only preying upon alates and completely avoiding workers (which on turn, behaved aggressively biting the geckos and often temporarily repelling them).

## 4 Discussion

### 4.1 Nest Sites, Densities, and Population Sizes

So far, one comprehensive study about nesting preferences in *Ca. lateralis* was published (Vesnić et al. 2017). Vesnić et al. (2017), who performed the study in the Western Balkans, found 94% of 49 nests in different kinds of wood (in our study also 94% of 142 nests were in wood) and only 6% in soil under stones. Other authors (Zimmermann 1935; Menzel, Woywod, et al. 2010; Seifert 2018) found the nests only or mainly in the soil (under stones)—but they had a smaller sample size (e.g., 6 nests in Menzel, Woywod, et al. 2010). In Vesnić et al. (2017) and in our study, respectively, 16% and 2% of nests were arboricolous. Nesting in wood could have acted as an evolutionary facilitator for the association with the arboricolous *Crematogaster* models. Although the ecological study of Vesnić et al. (2017) showed interesting similarities to our findings, it did not address any question regarding the ecological factor “*Crematogaster*”—in our opinion, a central factor determining the presence or absence of *Ca. lateralis* (see Section 4.4 | Putative associations with *Crematogaster*).

While the mean nest of *Ca. lateralis* contained little over 100 workers, and we estimate it is similar in *Ca. dalmaticus* and *Ca. rebeccae*, we do not know in how many cases nests represented the whole colony or only a part of it. Based on the spatial distribution of nests, we suggest the majority of colonies were monogynous–monodomous, and some nests were weakly polygynous with 2 queens or weakly polydomous with two nests in branches close to each other. The situation in *Ca. piceus* is similar (Borovsky 2018). The largest nest of *Ca. lateralis* contained ca. 700 workers (main site 15). Since we are not aware of a single colony exceeding 1000 workers, which is in line with some literature (Rachid et al. 2004; Stukalyuk and Radchenko 2011; Lebas et al. 2016; Radchenko 2016; Borovsky et al. 2022), we consider Seifert's (2018, 262) report of “10,000 workers” for “the largest nest in captivity” as not occurring under natural conditions. A small colony size—in relation to the much larger colonies of the *Crematogaster* model species—might be important for the evolution of mimicry because being in the minority is a criterium for evolving Batesian mimicry (Wickler 1968; Kikuchi and Pfennig 2010). Literature data do not give any reason to consider the colonies of *Ca. lateralis* as smaller than those of related species: Lebas et al. (2016) estimated that all species of the *Ca. lateralis* and the *Ca. kiesenwetteri* groups have a few dozen to a few hundred workers, and Seifert (2008, 2017, 2018) several hundreds to over a thousand for the *Ca. lateralis* group. Hence, we consider the small colony sizes of *Ca. lateralis* as preadaptation for the evolution of mimicry.

### 4.2 Trail‐Following Behavior

We observed nine species of *Camponotus* and *Colobopsis* following at least occasionally pheromone trails of *Crematogaster*. *Colobopsis imitans*, studied by Schifani et al. (2022, 2024), is a tenth Mediterranean example. Such a high number of species following *Crematogaster* trails is surprising, as literature reports with similar observations are largely lacking. Only in *Ca. lateralis*, trail following has been rather well investigated (Goetsch 1942, 1951, 1953; Kaudewitz 1955; Baroni Urbani 1969; Menzel, Woywod, et al. 2010; Stukalyuk et al. 2023). The published data on such behavior for “*Ca. piceus*”, *Ca. spissinodis*, *Co. truncata*, and *Co. imitans* are rather rare (Forel 1898; Goetsch 1942, 1951; Baroni Urbani 1969; Kutter 1977; Wagner 2019; Schifani et al. 2022, 2024). The material corresponding to the report about *Ca. piceus* (Schifani et al. 2022), however, belongs to *Ca. spissinodis*, based on our recent morphometric investigation. Our observations, together with literature data, suggest that the behavior is common in *Ca. lateralis*, *Ca. kiesenwetteri*, and *Colobopsis* spp., and rare in *Ca. dalmaticus*, *Ca. rebeccae*, *Ca. piceus*, *Ca. ruber*, and *Ca. spissinodis*. In *Ca. gestroi* in Greece, it appears to be moderately frequent. It has already been suggested that compounds used as trail pheromones by *Crematogaster* are generally easily perceived by *Camponotus*; this ability represents an important preadaptation to trail following (Menzel et al. 2014).

In most documented cases, interspecific trail sharing and mimicry occur together, while cases of ant mimicry without reports of trail sharing are rare (Table A1). The trail‐following observations of non‐mimetic species of the *Ca. lateralis* group *sensu* Seifert (2019) as well as *Ca. kiesenwetteri* are essential for understanding the evolutionary history of mimicry in *Ca. lateralis*. Since this behavior occurs in several non‐mimetic species (e.g., *Ca. kiesenwetteri*), it is most parsimonious to expect trail‐following to evolve first and mimicry later. It is possible that intermingling among *Crematogaster* workers without performing color mimicry (Goetsch 1942, 1951; Schifani et al. 2022) already reduces predation. If we consider trail following as the starting position, then each slight step towards visual mimicry could be adaptive. The fact that trail following occurs, on a worldwide scale, in several *Camponotus* subgenera (Ito et al. 2004; Menzel et al. 2014), in *Colobopsis* (Goetsch 1942; Baroni Urbani 1969; Wagner 2019; Schifani et al. 2022, 2024), and in *Polyrhachis* (Gobin et al. 1998), indicates that the whole tribe Camponotini has an ancestral tendency to evolve trail‐following behavior.

Workers of all observed trail‐following species were in the minority compared to those of *Crematogaster* on the trail, likely due to much smaller colony sizes of the trail‐following species. The percentage in *Ca. lateralis* was only 2.4% of that of *Crematogaster* and even smaller in all other trail‐following species. Being in the minority is in line with the theoretical underpinning of Batesian mimicry (McIver and Stonedahl 1993). Theory predicts that mimicry is most effective when the model is relatively more abundant (Wickler 1968; Huheey 1984; Lindström et al. 1997; Kunte et al. 2021).

The underrepresentation of major *Camponotus* workers on *Crematogaster* trails, compared with minor workers, has already been recognized (Goetsch 1942; Stukalyuk et al. 2023). This is also consistent with the theory of mimicry, as only the minor workers mimic *Crematogaster* in size, while major workers are distinctly larger (Stukalyuk et al. 2023). At first glance, this gives the impression of representing an adaptation to mimicry. However, the foraging activity of majors is equally reduced in the related non‐mimetic species *Ca. dalmaticus* and *Ca. piceus* (Seifert 2018). Moreover, our results suggest that the portion of major foragers on *Crematogaster* trails in *Ca. lateralis* is similar to that in the non‐mimetic *Ca. kiesenwetteri*. Also in the non‐closely related *Ca. foreli* Emery, 1881, minor workers are foragers while majors often stay in the nest (Espadaler et al. 1990). That foraging workers of *Ca. lateralis* are mainly minors, while major workers mainly stay in the nest, is an ancestral characteristic and probably a preadaptation for mimicry but not a mimicry‐specific adaptation.

### 4.3 No Color‐Based Aggression in *Crematogaster*

Baroni Urbani (1969) stated that the association of *Co. truncata* with *Cr. scutellaris* was similar or identical to that of *Ca. lateralis*. In contrast, Goetsch (1942, 1951) argued that the aggression of *Cr. scutellaris* was higher against *Co. truncata* than against *Ca. lateralis*. Our data (Table 2) do not support the latter statement. Concerning the observations of most authors and us, all Mediterranean species of the *Cr. scutellaris* group behave aggressively against *Ca. lateralis* (Baroni Urbani 1969; Marlier et al. 2004; Carpintero et al. 2005; Stukalyuk et al. 2023), but some kind of shift to tolerance suggests that *Cr. scutellaris* can habituate to *Ca. lateralis* (Kaudewitz 1955; Menzel, Woywod, et al. 2010). *Camponotus lateralis* and *Co. imitans* are known to exploit their higher speed to escape from *Crematogaster* (Baroni Urbani 1969; Schifani et al. 2022; Stukalyuk et al. 2023). It has been suggested that larger eyes and longer antennae contribute to the fast reaction of *Ca. lateralis* to escape from attacks of *Crematogaster* (Stukalyuk et al. 2023). Since these morphological traits and the high speed are present in other species of the *Ca. lateralis* group (for scape length of 11 Mediterranean species see in Seifert 2019), we consider them as a preadaptation to trail‐following behavior. The aggressiveness of *Crematogaster* workers, which are always in the majority in mixed trails, seems to pose a constant threat that *Camponotus* or *Colobopsis* workers are preadapted to cope with. The fact that *Camponotus* or *Colobopsis* workers of the same colony, which met each other on or near a *Crematogaster* trail, escaped from each other, reflects their high sensitivity. It might be cheaper to escape erroneously from a non‐existing *Crematogaster* worker than, vice versa, to miss escaping from an existing one.

Video records did not give any hint that *Crematogaster* workers treated non‐mimetic *Camponotus* species (*Ca. dalmaticus*, *Ca. gestroi*, *Ca. kiesenwetteri*, *Ca. piceus*, *Ca. rebeccae*, and *Ca. spissinodis*) differently from *Ca. lateralis* as well as abnormal colored *Crematogaster* workers differently from normal nestmates. It is meanwhile known that recognition of nestmates in ants is largely based on chemical recognition cues (cuticular hydrocarbons) (Hölldobler and Kwapich 2022 and citations herein). The same applies to *Crematogaster* workers to distinguish nestmates from *Camponotus* workers (Menzel, Blüthgen, and Schmitt 2008; Menzel et al. 2009; Menzel, Woywod, et al. 2010; Menzel and Schmitt 2012). Hence, based on our results and the mentioned literature, we reject Goetsch's (1942, 1951) and Kaudewitz' (1955) hypothesis about the ability of *Crematogaster* to discriminate nestmates via color and, consequently, also the hypothesis that *Ca. lateralis* exhibits aggressive mimicry (hypothesis iv).

### 4.4 Putative Associations With *Crematogaster*

The majority of *Ca. lateralis* nests (79%) was associated with *Crematogaster*, which gives the impression that this association is more important for the mimic than previously thought. Seifert's (2018) estimated frequency of this association of up to 20%, and similarly of ca. 28% in the study by Kaudewitz (1955), is much lower than what was shown in our study. It should be, however, added that our high percentage value holds true only for near‐natural biotopes. Our observations did not take place in strongly anthropogenically influenced sites since they do not reflect the environment in which the mimicry evolution in *Ca. lateralis* took place. Our previous, mostly occasional observations of *Ca. lateralis* at less structured sites, for example in gardens or at single trees at parking spots, revealed rarer associations with *Crematogaster*, simply because less structural diversity means less chance of the establishment of large *Crematogaster* colonies.

In the literature of the 19^th^ and 20^th^ century, when most observations were made in Italy or Spain, only *Cr. scutellaris* was mentioned as the association partner of *Ca. lateralis* (Gené 1842; Emery 1886; Goetsch 1942, 1951, 1953; Kaudewitz 1955; Baroni Urbani 1969). In newer literature, *Cr. schmidti* (Stukalyuk and Radchenko 2011; Stukalyuk et al. 2023) and rarely *Cr. ionia* (Wagner 2014; Salata et al. 2020) were also mentioned. Our data suggest that all Mediterranean species of the *Crematogaster scutellaris* group, *Cr. scutellaris*, *Cr. schmidti*, *Cr. ionia*, and *Cr*. cf. *ionia* sp. 1, have the same suitability for the association.

We found a positive correlation between the association of *Ca. lateralis* with *Crematogaster* and its worker number as well as with its probability to produce sexuals. To interpret this relation as causality (and not as a consequence of preferring sites with the same ecological conditions like suitable temperature or being rich in food), it seems likely that *Ca. lateralis* nests putatively associated with *Crematogaster* had a higher fitness as a result of this association itself. We suggest three probable positive influences of which two have already been discussed by Menzel, Woywod, et al. (2010) and others:

1. Locating food resources (Goetsch 1942, 1951, 1953; Menzel, Woywod, et al. 2010; Stukalyuk et al. 2023): Workers follow the pheromone trails of *Crematogaster* to reach food resources like aphid colonies. During our observations, *Camponotus lateralis* was observed together with *Crematogaster* spp. with honeydew‐producing hemipterans on *Hedera helix* , *Quercus*, *Pinus*, *Eucalyptus*, *Olea*, and *Ficus* trees. In one case each, both *Cr. scutellaris* and *Ca. lateralis* workers were observed together biting into lizard feces (Video 2 in Supplement) and bird feces. This explanation is valid also for all those trail followers which do not perform mimicry (e.g., *Ca. kiesenwetteri*). Hence, it is a primary evolutionary reason for the association.
2. Avoidance of predation by Batesian mimicry (Emery 1886; Collingwood 1993; Wagner 2013, 2014, 2025): Mimicry, presumably to reduce predation, is realized among Mediterranean ants only in *Ca. lateralis*, *Ca. ruber*, and *Colobopsis* spp. (Schifani et al. 2022) and is thus a derived strategy.
3. Protection from aggressive ant competitors: As a third advantage conveyed by proximity to *Crematogaster* a putatively reduced occurrence of dangerous ant competitors should be tested. For example, aggressive species like *Lasius emarginatus* (Olivier, 1792) and *L. niger* (Linnaeus, 1758) (Seifert 2018) can be life‐threatening for *Ca. lateralis* (Borovsky et al. 2022). We experimentally brought a branch with a part of a *Cr. schmidti* nest close to a *L. emarginatus* nest and observed that workers of the latter—although in the majority—kept distance from those of the former (Video 32 in Supplement). Moreover, on trees, where a species of the *Cr. scutellaris* group dominates, it is unlikely to find other numerous and aggressive ant species (Stukalyuk and Radchenko 2011; we never found aggressive *Lasius* spp. nesting on the same tree as *Crematogaster*). Hence, territories of *Crematogaster* might be protected areas for *Ca. lateralis* but putatively also for related *Camponotus* species.

Vice versa, we have no hint that *Crematogaster* has any benefit from being associated with possible food competitors like *Ca. lateralis*. Moreover, mimetic species of *Camponotus* or *Colobopsis* should cause a dilution effect of the aposematic signal which might lead to error attacks of predators against *Crematogaster* (cf. Kikuchi and Pfennig 2013). Empirically, the permanent attacks against *Camponotus* and *Colobopsis* workers (see Section 4.3 | No color‐based aggression in *Crematogaster*) on *Crematogaster* trails make their undesirability obvious. We, consequently, expect that *Crematogaster* rather suffers than benefits from the association and selection should favor strategies to get rid of mimics.

The strong positive relation between *Crematogaster* presence and worker number as well as sexual production leads to the impression that some kind of dependence occurs. Consistent with this putative dependence are distribution data, available in the literature: Localities where *Ca. lateralis* occurs without *Crematogaster* are exceptions (Carpintero et al. 2001, 2005; Ordóñez‐Urbano et al. 2007; Santini et al. 2007; Gratiashvili and Barjadze 2008; Bračko et al. 2014, 2016; Borowiec and Salata 2017, 2018; Bračko 2019, 2023; Kiran and Karaman 2020; Salata et al. 2020; Borowiec et al. 2021, 2024). According to maps in Lebas et al. (2016, 139, 273, 275), species of the *Cr. scutellaris* group and *Ca. lateralis* have a nearly identical distribution. Menzel et al. (2014) considered *Ca. lateralis* to be independent of *Crematogaster*. However, data about the ecological niche, worker numbers, sexual production, and distribution of *Ca. lateralis* suggest that stable populations without *Crematogaster* do not exist. Theory predicts that mimicry is most effective when the range of the mimic is embedded by that of the model, so that predators encountering the mimic are likely to have prior experience with the model (Wickler 1968; Pough 1987).

### 4.5 Association With *Crematogaster* in *Camponotus* and *Colobopsis* Gynes

We found gynes of *Camponotus lateralis* and of *Ca. kiesenwetteri* following *Crematogaster* trails and visiting wood openings in their surroundings and close to their nests. We interpret this behavior as an attempt to establish colonies in proximity to *Crematogaster*. Menzel, Woywod, et al. (2010) found dealate *Ca. lateralis* gynes frequently under rocks at less than 1 m distance to *Cr. scutellaris* nests, but not farther away from them. Baroni Urbani (1969) mentioned the attraction of *Ca. lateralis* gynes for *Crematogaster* trails and selecting a site next to *Crematogaster* to establish their colonies. He also observed a dealate gyne of *Colobopsis truncata* following a trail of *Cr. scutellaris*, while we observed a gyne following a *Dolichoderus quadripunctatus* trail (video 26 in Supplement). In strong contrast, Kaudewitz (1955) argued that the low frequency of *Ca. lateralis* nests in associations with *Crematogaster* indicates against the preference of gynes to establish colonies in proximity to *Crematogaster*. Most likely, he underestimated the percentage of associated nests in near‐natural biotopes due to collecting in anthropogenic biotopes (see Section 4.4 | Putative associations with *Crematogaster*). It may seem surprising that *Ca. kiesenwetteri* gynes attempt to establish new colonies in wood, since it is known that this species nests in soil, most often under trees (Salata et al. 2019). We assume that a nest relocation follows as soon as the population reaches a certain size. To summarize, at least three trail‐following species, *Ca. lateralis*, *Ca. kiesenwetteri*, and *Co. truncata* select founding chambers next to *Crematogaster* trails or nests.

### 4.6 Defending Nest Entrances Against *Crematogaster*

While *Camponotus lateralis* was submissive and escaped or sidestepped if meeting a *Crematogaster* worker on a trail, we observed an inverse dominance hierarchy at one nest entrance. Nests of *Ca. lateralis* have only a few small entrances which can be blocked by major workers with their heads against *Cr. schmidti*. The majority of *Crematogaster* workers avoided the nest entrance of *Ca. lateralis* after antennal contact. Since the *Cr. schmidti* workers were provoked to aggression by the collector, we expect an even lower attack rate under natural conditions. While the proximate explanation for this aversion should be searched in the recognition of any chemical substance, the ultimate explanation remains enigmatic: How should a substance, indicating the presence of a few tens of major workers of *Ca. lateralis*, represent an honest power signal against thousands of *Crematogaster* workers? In any case, the nest‐defending strategy, a combination of blocking behavior and possible use of aversive chemicals, might turn out to be a key explanation why *Ca. lateralis* can live in proximity to *Crematogaster*—a niche which enables mimicry. Since we do not know if this behavior of *Ca. lateralis* is unique within the *Ca. lateralis* group, we cannot decide if it evolved before or after mimicry. It was, however, noted that also in the non‐closely related *Camponotus aethiops* (Latreille, 1798) large workers seldom forage and seem to be specialized for nest guarding (Laffort et al. 1991). In many *Camponotus* species, major workers have also a military function (Lamon and Topoff 1981; Busher et al. 1985; Espadaler et al. 1990). We observed only a few *Crematogaster* workers besmearing the *Ca. lateralis* majors at the nest entrance with their venom. It has been reported that this venom is lethal for *Lasius niger* and *Pheidole pallidula* (Nylander, 1849) but not for *Ca. lateralis* (Marlier et al. 2004). Marlier et al. (2004) already hypothesized that *Ca. lateralis* has evolved some specific mechanisms of resistance to the venom of *Cr. scutellaris* due to its close association.

In *Colobopsis truncata*, phragmosis, an adaptation of major workers to block nest entrances against hostile ants (Wheeler 1927; Dumpert 1978), is well known. The plug‐like truncate shape of the heads of majors represents a special adaptation for this behavior (Brun 1924; Stitz 1939; Goetsch 1953; Hölldobler and Wilson 1990; Seifert 2008, 2018). The possibility of effectively blocking nest entrances against *Crematogaster* has already been suggested (Goetsch 1942, 1951). In *Ca. lateralis*, this morphological adaptation to phragmosis is completely absent—despite the similar behavior. It seems possible that *Ca. lateralis* is at an earlier evolutionary stage concerning this nest‐defending strategy than *Colobopsis*. Thus, we term the strategy in *Ca. lateralis* “pre‐phragmosis”.

### 4.7 Mimicry

It has been hypothesized that *Ca. lateralis* represents regional morphs mimicking the color patterns of three *Crematogaster* model species (Wagner 2014; Seifert 2018, 2019): (i) *Crematogaster scutellaris* with a reddish head and a blackish mesosoma, (ii) *Cr. schmidti* with a reddish head and a reddish mesosoma, and (iii) *Cr. ionia* (and *Cr*. cf. *ionia* sp. 1) with a brownish to blackish head and mesosoma. This hypothesis is currently investigated using color measurements (Kraker and Wagner 2025); here we briefly discuss our obvious novelties. On Crete, where *Cr. scutellaris* and *Cr. schmidti* are absent (Salata et al. 2020), nearly all *Ca. lateralis* workers were dark brown to blackish, sometimes with a head slightly lighter than the rest of the body, resembling the color of *Cr. ionia* and *Cr*. cf. *ionia* sp. 1.

The situation regarding color morphs seems to be similar in Mediterranean *Colobopsis* species. For the recently described *Co. imitans*, a *Cr. scutellaris* mimicry has already been shown (Schifani et al. 2022), while *Co. truncata* mimics *Dolichoderus quadripunctatus* (Forel 1874; Wagner 2019; Schifani et al. 2022). For the Cretan population of *Colobopsis*, whose species identity is not fully clear yet, we found only dark brownish to blackish minor workers, putatively mimicking the brownish to blackish Cretan *Crematogaster ionia* and *Cr*. cf. *ionia* sp. 1.

*Camponotus ruber* has been considered to be a mimic of *Cr. scutellaris* (Schifani et al. 2022). The two species coexist in the same biotops in both Sicily and Tunisia, with *Ca. ruber* nests found in the soil and under stones. However, its color pattern appears to be a less accurate form of mimicry of *Cr. scutellaris* compared to the often syntopic *Ca. lateralis*. Furthermore, the trail‐following behavior is perhaps as rare as in most non‐mimicking species. In the phylogenetically closely related and also mimetic species *Ca. guanchus*, following of *Crematogaster* trails is also rare (Pérez‐Delgado and Wagner 2024).

*Camponotus rebeccae* has a similar color pattern as *Ca. lateralis* (Seifert 2019). The reddish color of *Ca. rebeccae* workers at three localities on Crete is—in contrast to those of *Ca. lateralis*—clearly different from regional *Cr. ionia* and *Cr*. cf. *ionia* sp. 1 workers. Hence, this species can be excluded as representing a mimic of *Crematogaster*.

In *Ca. gestroi*, while a high similarity in terms of color was given with *Crematogaster* on Crete, workers in western Greece (main site 12) in sympatry with *Cr. schmidti* as well as on Sicily in sympatry with *Cr. scutellaris* were also fully blackish. Hence, we do not consider it to be a mimic.

Workers of all species of the *Ca. lateralis* group have a smooth cuticula surface (Seifert 2019), similar to that of workers of the *Crematogaster scutellaris* group. *Camponotus kiesenwetteri* , although its trail‐following behavior is as well pronounced as in *Ca. lateralis*, is larger in size, homogenously gray, and has a dull cuticula surface with dense pubescence (Lebas et al. 2016; Salata et al. 2019). Hence, it exhibits a distinct optical contrast to *Crematogaster* (Video 8 in Supplement), which makes us believe that size, color, or cuticula‐surface modifications in the direction of the model would not bring any selective advantage and, consequently, visual mimicry could not evolve.

The size of *Ca. lateralis* minor workers is similar to those of the *Crematogaster* model‐species. Seifert (2018) gives for *Ca. lateralis* workers (majors included) a mean head index of CS = 1278 μm. Further six species of the *Ca. lateralis* group show CS values of 1140 to 1318 μm (Seifert 2019). Since *Ca. lateralis* does not significantly differ from related species, no species‐specific mimicry adaptation occurs in size.

### 4.8 Field Observations on Predators

Our field observations provide at least some support for the idea that lizards drive the evolution of mimicry (Pérez‐Delgado and Wagner 2024; Wagner 2025). Although in all of our presented anecdotes a species of the *Cr. scutellaris* group was common at sites with lizards, only in one case was *Crematogaster* ingested by a *Podarcis siculus* lizard. The observation of a young male of *P. siculus* at main site 5b is interesting and its behavior of touching *Cr. scutellaris* with its tongue but not ingesting it can be interpreted as follows: The lizard already had prior experience with both ant species, *Cr. scutellaris* and *Ca. lateralis*; since it was not able to identify the worker visually, it tasted it with the tongue and, since it was unpalatable, avoided it. An adult female of *Algyroides nigropunctatus* ignored several workers of *Cr. schmidti* but ingested a gyne of *Ca. dalmaticus* while workers of *Cataglyphis* and *Ca. dalmaticus* escaped after recognizing the lizard. Not only mimicry in *Ca. lateralis*, but also the escaping reaction of *Cataglyphis* and *Ca. dalmaticus* after the perception of the lizard can be interpreted as adaptation against predation. Two adults of *P. erhardii* ingested *Ca. dalmaticus* major workers in the field. The observations are in line with laboratory and field investigations of *P. siculus* : An adult male ingested > 40 workers of *Ca. lateralis* or *Ca. piceus* per day but avoided *Cr. scutellaris* after tasting in the laboratory (Wagner 2013, 2014, 2025); moreover, a recent study on feces of *P. muralis* found that *Cr. scutellaris* and *Ca. lateralis* were highly significantly underrepresented consumed, in comparison with other ants (e.g., *Ca. piceus*). This finding clearly supports our hypothesis that lizards avoid *Crematogaster* because of its bad taste and aposematism and *Ca. lateralis* because of Batesian mimicry (Wagner 2025).

Summarizing the results, we consider our anecdotes as small hints that lizards of the genera *Podarcis* and *Algyroides* ingest ants but avoid *Crematogaster*. Laboratory experiments will test the hypothesis of mimicry in *Ca. lateralis*. The comparison of lizard and *Ca. lateralis* nest densities at our 19 main sites (Tables A5 and A6) results in a ratio of about seven nests to one lizard on average. If one nest contains 105 workers and one lizard can ingest > 40 workers per day, it has the hypothetical possibility to exterminate one *Ca. lateralis* nest within 3 days and all nests of its territory in 1 month. Consequently, a high selection pressure on *Ca. lateralis* to mimic *Crematogaster* should be given.

Geckos may potentially also have a role as ant predators; however, since they lack red color perception (Loew 1994), they cannot be considered drivers of color‐mimicry evolution. In any case, our observations signal the unpalatability of workers of *Cr. scutellaris* to these predators too.

## 5 Assessment of Hypotheses i–iv

We found that workers of *Ca. lateralis* were much less frequent than those of *Crematogaster* (see Section 4.1 | Nest sites, densities, and population sizes and Section 4.2 | Trail‐following behavior); being distinctly in the minority is a criterium for Batesian mimicry, but it is untypical for Müllerian mimicry (Wickler 1968; Kikuchi and Pfennig 2010). Workers of *Ca. lateralis* intermingle among those of *Crematogaster* while, vice versa, *Crematogaster* behaves aggressively against *Ca. lateralis* (Section 4.3 | No color‐based aggression in *Crematogaster*). Whereas seeking spatial proximity should be adaptive for a Batesian mimic, selection should favor the model avoiding such proximity to reduce the dilution of an aposematic signal (Kikuchi and Pfennig 2013). Moreover, species of the *Cr. scutellaris* group are avoided by lizards, while those of the *Ca. lateralis* group are consumed (Section 4.8 | Field‐observations on predators), which ranks the mimicry of *Ca. lateralis* as Batesian and rejects Müllerian mimicry (Emery 1886; Menzel, Woywod, et al. 2010; Menzel et al. 2014; Seifert 2019; Schifani et al. 2022; Pérez‐Delgado and Wagner 2024; Wagner 2025). Hypothesis iii, the dilution effect, cannot be excluded from playing any role in addition to Batesian mimicry concerning some predators. Our argumentation that predation drives mimicry in *Ca. lateralis* makes aggressive mimicry (Goetsch 1942, 1951; Kaudewitz 1955), which was rejected by our behavioral analyses (see Section 4.3 | No color‐based aggression in *Crematogaster*), dispensable as an alternative explanation.

## 6 Conclusions on the Evolution of Mimicry in *Camponotus lateralis*

It has already been suggested that the genera *Camponotus* and *Crematogaster* possess life‐history traits that favor the evolution of associations. Menzel et al. (2014) listed three preadaptations, of which two at least partly hold true also for Mediterranean species: *Camponotus* can perceive trail pheromones of *Crematogaster* and there are parallel shifts in cuticular‐hydrocarbon profiles between *Camponotus* and *Crematogaster* species. However, the similarity of cuticular hydrocarbons apparently does not lead to tolerance of *Ca. lateralis* by workers of the *Cr. scutellaris* group (see also Menzel, Woywod, et al. 2010).

We found that most characteristics beneficial for mimicry in *Ca. lateralis* are obviously not unique to this mimetic species (Table 4). Hence, we believe that there are many prerequisites that enabled the evolution of color mimicry in *Ca. lateralis*. Such putative preadaptations in behavior, morphology, colony structure, and habitat selection can be common among Camponotini (e.g., high speed, large eyes, long antennae, perceiving *Crematogaster* trail‐pheromones) or specific to some *Camponotus* groups (size of minor workers, shiny cuticula surface, foraging workers being mostly minors, small colony sizes). Surprisingly, unique characteristics in *Ca. lateralis* are rare and only the manifestation of color morphs is an obvious mimicry adaptation.

**TABLE 4:** Main mimicry‐(pre)adaptations of Mediterranean *Camponotus* and *Colobopsis* species that follow trails of the *Crematogaster scutellaris* group (or of *Dolichoderus quadripunctatus* ).

	Nests in wood	Trail following	*Crematogaster*‐like size of minors	Shiny cuticula surface	Colony foundation near *Crematogaster*	(pre‐)phragmosis	Color mimicry
*Ca. dalmaticus*	Yes	Rare	Yes	Yes	No	Unknown	No
*Ca. gestroi*	No	Moderate	Yes	Yes	Unknown	Unknown	No
*Ca. kiesenwetteri*	Foundation: yes; later: no	Common	No	No	Yes	Unknown	No
*Ca. lateralis*	Yes	Common	Yes	Yes	Yes	Yes	Yes
*Ca. piceus*	No	Rare	Yes	Yes	No	Unknown	No
*Ca. rebeccae*	Yes	Rare	Yes	Yes	Unknown	Unknown	No
*Ca. ruber*	No	Rare	Yes	Yes	Unknown	Unknown	Yes
*Ca. spissinodis*	No	Rare	Yes	Yes	Unknown	Unknown	No
*Co. imitans*	Yes	Common	Yes	Yes	Expected	Yes	Yes
*Co. truncata*	Yes	Common	Yes	Yes	Yes	Yes	Yes

## Author Contributions

**Herbert C. Wagner:** conceptualization (lead), data curation (lead), formal analysis (lead), funding acquisition (lead), investigation (lead), methodology (lead), project administration (lead), resources (lead), software (lead), supervision (lead), validation (lead), visualization (equal), writing – original draft (lead), writing – review and editing (equal). **Felix Kraker:** data curation (supporting), investigation (supporting), visualization (equal), writing – review and editing (equal). **Gregor Bračko:** data curation (supporting), investigation (supporting), visualization (equal), writing – review and editing (equal). **Enrico Schifani:** data curation (supporting), investigation (supporting), visualization (equal), writing – original draft (supporting), writing – review and editing (equal).

## Funding

This research was funded in large part by the Austrian Science Fund (FWF) [10.55776/P35816]. For the purpose of open access, the author has applied a CC BY public copyright license to any Author Accepted Manuscript version arising from this submission.

## Conflicts of Interest

The authors declare no conflicts of interest.

## Data Availability

The Supplement videos that support the findings of this study are on https://figshare.com/s/b627084f6a7f60e6dc4c.

## Appendix A

**TABLE A1:** Worldwide list of non‐social‐parasitic ant species applying trail sharing and/or (Batesian) ant mimicry as well as one case of termite mimicry. Putative examples of Müllerian mimicry in squared brackets.

Trail follower/mimic	Trail layer/model	Trail sharing	(Batesian) mimicry	Literature
*Atta mexicana* (Smith, 1858)	*Acromyrmex versicolor* (Pergande, 1893)	Yes	No	Mintzer (1980)
*Axinidris palligastrion* Shattuck, 1991	*Crematogaster clariventris* Mayr, 1895	Yes	Yes	Fisher and Bolton (2016)
[ *Camponotus aeneopilosus* Mayr, 1862]	*Polyrhachis appendiculata* Emery, 1893, *P. ammon* (Fabricius, 1775), *P. argentosa* Forel, 1902	Unknown	Yes	Pekár et al. (2017)
*Camponotus beebei* Wheeler, 1918	*Azteca chartifex* Forel, 1896	Yes	Unknown	Wilson (1965)
*Camponotus blandus* (Smith, 1858)	*Pseudomyrmex termitarius* (Smith, 1855)	Yes	Yes	Gallego Ropero and Feitosa (2014)
*Camponotus concurrens* Zettel & Laciny, 2018	*Colobopsis cylindrica* (Fabricius, 1798)	Yes	Yes	Zettel et al. (2018)
*Camponotus dalmaticus* (Nylander, 1849)	*Crematogaster schmidti* (Mayr, 1853)	Yes	No	This study
*Camponotus femoratus* (Fabricius, 1804)	*Crematogaster carinata* Mayr, 1862, *Cr. levior* Longino, 2003	Yes	No	Wheeler (1921); Weber (1943); Swain (1980); Vantaux et al. (2007); Vicente and Izzo (2017)
*Camponotus gestroi* Emery, 1878	*Crematogaster ionia* , *Cr*. cf. *ionia* sp. 1, *Cr. schmidti*	Yes	No	This study
*Camponotus guanchus* Santschi, 1908	*Crematogaster alluaudi* Emery, 1893	Yes	Yes	Emery (1893); Santschi (1919); Pérez‐Delgado and Wagner (2024)
*Camponotus imitator* Forel, 1891	*Aphaenogaster swammerdami* Forel, 1886	Yes	Yes	Forel (1898); Rasoamanana et al. (2017); Csősz et al. (2021); unpublished observations of B. Fisher
*Camponotus inquilinus* Zettel & Laciny, 2018	*Colobopsis* sp. “nrSA” *sensu* Laciny et al. (2017)	Yes	Yes	Zettel et al. (2018)
*Camponotus jodina* Rasoamanana et al. 2017	*Aphaenogaster swammerdami* group	Yes	Yes	Rasoamanana et al. (2017); Csősz et al. (2021)
*Camponotus karaha* Rasoamanana et al. 2017	*Aphaenogaster swammerdami* group	Yes	Yes	Rasoamanana et al. (2017); Csősz et al. (2021)
*Camponotus kiesenwetteri* (Roger, 1859)	*Crematogaster ionia* , *Cr*. cf. *ionia* sp. 1	Yes	No	This study
*Camponotus lateralis* (Olivier, 1792)	*Crematogaster scutellaris* (Olivier, 1792), *Cr*. *schmidti* (Mayr, 1853), *Cr*. *ionia* Forel, 1911, *Cr*. cf. *ionia* sp. 1	Yes	Yes	e.g., Emery (1886); Forel (1898); Goetsch (1942); Goetsch (1951); Goetsch (1953); Kaudewitz (1955); Baroni Urbani (1969); Carpintero et al. (2005); Menzel, Woywod, et al. (2010); Stukalyuk et al. (2023); this study
*Camponotus longicollis* Rasoamanana et al. 2017	*Aphaenogaster swammerdami* group	Yes	Yes	(Rasoamanana et al. 2017; Csősz et al. 2021)
*Camponotus piceus* (Leach, 1825)	*Crematogaster scutellaris* (Olivier, 1792)	Yes	No	This study
[ *Camponotus pulchellus* Forel, 1894‐complex], *Ca. irritans croceomaculatus* Emery, 1914	*Rhytidoponera mimica* Ward (1984)	Unknown	Yes	Ward (1984); P. S. Ward in litt.
*Camponotus putatus* Forel, 1892 complex species *sensu* Ward (2009)/*Camponotus* sp. *sensu* Fisher & Peeters (Fisher and Peeters 2019)	*Tetraponera grandidieri* (Forel, 1891)	Yes	Yes	Ward (2009); Fisher and Peeters (2019)
*Camponotus reaumuri* Forel, 1892	*Tetraponera inermis* (Ward 2009)	Unknown	Yes	Ward (2009)
*Camponotus rebeccae* Forel, 1913	*Crematogaster* cf. *ionia* sp. 1	Yes	No	This study
*Camponotus rufifemur* Emery, 1900	*Crematogaster modiglianii* Emery, 1900	Yes	No	Menzel, Linsenmair, and Blüthgen (2008); Menzel et al. (2014); Menzel and Blüthgen (2010)
*Camponotus* sp. *sensu* Ito et al. (2004)	*Crematogaster inflata* Smith, F., 1857	Yes	Yes	Ito et al. (2004)
*Cephalotes mariadeandrade* Oliveira et al. 2021	*Crematogaster* sp.	Yes	Yes	Oliveira et al. (2021)
*Cephalotes specularis* Brandão et al. 2014	*Crematogaster ampla* Forel, 1912	Yes	Yes	Brandão et al. (2014); Powell et al. (2014)
*Camponotus spissinodis* Forel, 1909	*Crematogaster scutellaris*	Yes	No	Schifani et al. (2022 sub *piceus*); Schifani et al. 2024; this study
*Camponotus suffusus* *bendigensis* Forel, 1902	*Myrmecia fulvipes* Roger, 1861	Unknown	Yes	Merrill and Elgar (2000)
*Camponotus trietericus* Menozzi, 1926	*Colobopsis corallina* Roger, 1863	Yes	Yes	Zettel et al. (2018)
[*Colobopsis camelus* (Emery, 1883)‐complex]	*Rhytidoponera mimica* Ward (1984)	Unknown	Yes	(Ward 1984; P. S. Ward in litt. 2021)
*Colobopsis imitans* Schifani et al., 2021	*Crematogaster scutellaris* (Olivier, 1792)	Yes	Yes	Carpintero et al. (2005 sub *Camponotus truncatus* ); Schifani et al. (2022)
*Colobopsis truncata* and *Co*. cf. *truncata*	*Dolichoderus quadripunctatus* (Linnaeus, 1771), *Crematogaster scutellaris* , *Cr. schmidti Cr. ionia*, *Cr*. cf. *ionia* sp. 1	Yes	Yes	Forel (1874); Goetsch (1942, 1951); Baroni Urbani (1969); Kutter (1977); Collingwood (1993); Carpintero et al. (2005); Wagner (2019); García (2020); Schifani et al. (2022); this study
*Dolichoderus debilis* Emery, 1890	*Crematogaster carinata* Mayr, 1862	Yes	No	Forel (1898); Wheeler (1921)
*Pheidole nasutoides* Hölldobler and Wilson (1992)	Termites of the genus *Nasutitermes*	No	Yes	Hölldobler and Wilson (1992)
*Polyrhachis rufipes* Smith, F., 1858	*Stictoponera menadensis* (Mayr, 1887)	Yes	Unknown	Gobin et al. (1998)

**TABLE A2:** Main sites (Figure 2) and additional sites (a1–a9).

Site	Locality	Coordinates	Biotope	Model	Date
1	Italy: 1.4 km WNE Passirano	45.6032° N, 10.0477° E, 252 m	Mixed‐deciduous‐forest margin (*Quercus*, *Fraxinus ornus* , *Castanea sativa* , *Robinia pseudoacacia* , *Sambucus nigra* , *Rubus*, *Hedera helix* )	*Cr. scutellaris*	24.–25.IV.2023
2	Italy: 1.2 km W Custoza	45.3702° N, 10.7798° E, 109 m	Deciduous‐forest remnant with *Quercus*, *Fraxinus ornus* , *Populus*, *Robinia pseudoacacia* , *Ailanthus altissima* , *Prunus*, *Ficus*, *Ulmus*, *Hedera helix* , *Smilax*. Steep east‐slope.	*Cr. scutellaris*	22.–23.IV.2023
3	Italy: 1.1 km NNW Battaglia Terme	45.2985° N, 11.7732° E, 14 m	*Morus* alley with *Hedera helix* and *Smilax*	*Cr. scutellaris*	21.–22.IV.2023
4a	Slovenia: 1.6 km NW Ankaran	45.5908° N, 13.7234° E, 18 m	*Quercus*‐*Ulmus* forest with *Hedera helix*	*Cr. schmidti*	17.–19.IV. and 15.X.2023
4b	Slovenia: 2.8 km NW Ankaran	45.5913° N, 13.7042° E, 17 m	*Pinus* forest with *Hedera helix*	*Cr. schmidti*	14.–15.X.2023
5a	Croatia: 3.8 km ESE Novigrad	45.3066° N, 13.6103° E, 69 m	Meadow with *Quercus* trees with *Hedera helix*	*Cr. scutellaris*	14.IV.2023
5b	Croatia: 3.3 km ESE Novigrad	45.3113° N, 13.6057° E, 16 m	*Pinus* forest in north exposition	*Cr. scutellaris*	15.IV.2023
6	Croatia: Krk, 1.7 km N Linardići	45.0889° N, 14.4667° E, 119 m	*Quercus*‐*Ulmus* forest with *Hedera helix*	*Cr. scutellaris*	12.–13.IV.2023, 12.–13.X.2023
7	Croatia: 5.5 km NW Pirovac	43.8476° N, 15.6123° E, 12 m	*Pinus* forest with *Hedera helix* and *Smilax*	*Cr. scutellaris*	9.–10.IV.2023
8	Croatia: 2.2 km NNW Makarska	43.3151° N, 17.0020° E, 69 m	*Pinus* forest	*Cr. scutellaris*	6.–7.IV.2023
9	Croatia: 9.5 km W Orobić	42.9851° N, 17.0676° E, 27 m	Macchia with *Smilax*	*Cr. schmidti*	4.–5.IV.2023
10	Montenegro: 3.8 km SE Petrovac	42.1836° N, 18.9759° E, 5 m	*Quercus* trees with *Hedera helix* along the street or single trees in gardens or fallow land	*Cr. schmidti*	2.–3.IV.2023
11	Albania: 8.0 km W Levan	40.6748° N, 19.3940° E, 1 m	*Pinus* forest with *Pistacia* and *Quercus* bushes	*Cr. schmidti*	26.–28.V.2023
12	Greece: 2.7 km SSE Ladochori	39.4685° N, 20.2476° E, 9 m	Deciduous forest (*Quercus coccifera*, *Olea europaea* , *Pistacia*, *Quercus ithaburensis* , *Genista*, *Smilax*). South‐west exposition	*Cr. schmidti*	15.–16.IV.2024
13	Greece: 2.0 km SW Araxos	38.1755° N, 21.3683° E, 2 m	*Quercus*‐*Pistacia* forest with *Smilax*	*Cr. schmidti*	12.–13.IV.2024
14	Greece: 2.2 km NNE Palea Epidauros	37.6582° N, 23.1631° E, 8 m	*Pinus* forest with *Pistacia* shrubs	*Cr. ionia*	9.–10.IV.2024
15	Greece: Crete, SW Lake Límni Agiás	35.4747° N, 23.9316° E, 43 m	*Platanus* forest with *Hedera helix*	*Cr. ionia*	4.–5.IV.2024
16	Greece: Crete, 1.4 km WSW Argiroupolis	35.2822° N, 24.3191° E, 180 m	Deciduous wood along river (*Platanus*, *Olea europaea* , *Quercus coccifera*, *Ceratonia siliqua* , etc.)	*Cr*. cf. *ionia* sp. 1 *sensu* Salata et al. (2020)	2.–3.IV.2024
17	Greece: Crete, 1.6 km WNW Limenas Chersonisou	35.3214° N, 25.3806° E, 25 m	*Eucalyptus* forest, occasionally *Phoenix theophrasti* and *Ficus*	*Cr*. cf. *ionia* sp. 1	31.III.–1.IV.2024
18	Greece: Crete, 0.8 km W Maronia	35.1404° N, 26.0728° E, 222 m	Humid *Eucalyptus*‐*Platanus* ditch with *Hedera helix* and *Ficus*	*Cr*. cf. *ionia* sp. 1	29.–30.III.2024
19	Greece: Karpathos, 5.7 km N Spoa	35.6877° N, 27.1504° E, 192 m	*Pinus* forest in north exposition	*Cr. ionia*	26.–27.III.2024
a1	Tunisia: 3.9 km SSW Cap Serrat	37.2067° N, 9.2321° E, 2 m	*Quercus* forest	*Cr. scutellaris*	29.IV.2024
a2	Tunisia: Kesra forest	35.8764° N, 9.4581° E, 836 m	*Pinus* forest	*Cr. scutellaris*	8.IV.2025
a3	Italy: Sicily, Palermo	38.1953° N, 13.3354° E, 5 m	Garden	*Cr. scutellaris*	Over years
a4	Austria: 2.0 km NN Zwaring	46.9269° N, 15.4026° E, 323 m	*Quercus*‐dominated broadleaved forest		21.VII.2024
a5	Slovenia: 1.4 km NE Dekani	45.5594° N, 13.8222° E, 302 m	Broad‐leaved forest	*Cr. schmidti*	24.VI.2024
a6	Slovenia: 0.1 km NE Osp	45.5722° N, 13.8597° E, 70 m	Broad‐leaved forest	*Cr. schmidti*	15.VI.2024
a7	Greece: Athens W Piräus	37.9658° N, 23.558° E, 16 m	Park with *Pinus* trees	*Cr. ionia*	25.III.2024
a8	Greece: Crete, 1.3 km E Souda	35.4855° N, 24.0891° E, 2 m	Shrubs on beach	*Cr. ionia*	7.IV.2024
A9	Greece: Crete, 0.5 km WNW Limne	35.2494° N, 25.6336° E, 207 m	Parking lot with *Pinus* trees	*Cr. ionia*	31.III.2024

**TABLE A3:** Nest types of *Camponotus lateralis* and two related species with similar ecology.

Nest type	*lateralis*	*dalmaticus*	*rebeccae*
Dead branches with 1–10 cm thickness lying on ground (mostly next to large trees)	90 (63%)	31 (82%)	2 (100%)
In dead standing stumps (which were connected via roots with the soil) < 125 cm above the ground	14 (10%)	3 (8%)	
Under bark or in wood of living trees on the trunk < 100 cm above ground	10 (7%)		
In dead branches leaning on trees or other plants 10–100 cm above ground	8 (6%)		
In dead rootstocks in the ground	5 (4%)		
In stone walls	4 (3%)		
In the soil (partly under stones; Video 2 in Supplement)	4 (3%)	2 (5%)	
Arboricolous on deciduous trees 100–250 cm above the ground	3 (2%)		
In lying logs with 10–20 cm thickness	3 (2%)		
Under palm leaves	1 (1%)		
In rotten wooden‐planks		1 (3%)	
Under concrete		1 (3%)	
Sum	142	38	2

**TABLE A4:** Tree species of wooden *Camponotus lateralis* nests.

*Quercus*	40
*Pinus*	39
*Platanus*	16
Unidentified shrubs	10
*Ailanthus altissima*	7
*Morus*	6
*Pistacia*	6
*Ulmus*	6
*Eucalyptus*	5
*Fraxinus*	5
*Olea*	4
*Sambucus*	3
*Prunus*	2
*Rubus*	2
*Ceratonia siliqua*	1
*Ficus*	1
*Hedera helix*	1
Palm tree	1
Reed	1
Thistle	1
Sum	157

**TABLE A5:** Nest densities of *Camponotus lateralis* , *Ca. dalmaticus*, and *Ca. rebeccae* at the main sites. The *n* is the number of counted *Ca. lateralis* nest‐populations per site.

Main site	Nest densities [per 100 m^2^] of three species			Worker number
	*lateralis*	*dalmaticus*	*rebeccae*	*lateralis*
1	< 0.5 nests			102 ± 167 (*n* = 5)
2	ca. 3 nests			42 ± 34 (*n* = 9)
3	0.5–1 nests			34 ± 29 (*n* = 10)
4a	1–2 nests			75 ± 62 (*n* = 6)
4b	1–2 nests			No data
5a	< 0.5 nests			54 ± 20 (*n* = 4)
5b	< 0.5 nests	0.5–1 nests		125 ± 0 (*n* = 2)
6	0.5–1 nests	0.5–1 nests		62 ± 39 (*n* = 7)
7	0.5–1 nests	0.5–1 nests		210 ± 151 (*n* = 5)
8	1–2 nests			100 ± 81 (*n* = 7)
9	No data	Common		99 ± 68 (*n* = 7)
10	1–2 nests	< 0.5 nests		90 ± 57 (*n* = 6)
11	2–3 nests	Rare		126 ± 96 (*n* = 6)
12	4–5 nests	10–11 nests		125 ± 30 (*n* = 4)
13	2–3 nests	1–2 nests		111 ± 78 (*n* = 8)
14	1–2 nests	< 0.5 nests		60 ± 33 (*n* = 6)
15	2–3 nests		< 0.5 nests	255 ± 304 (*n* = 4)
16	2–3 nests			218 ± 167 (*n* = 6)
17	0.5–1 nests			203 ± 25 (*n* = 3)
18	ca. 4 nests		ca. 2 nests	95 ± 61 (*n* = 3)
19	0.5–1 nests			111 ± 75 (*n* = 5)
Mean	1.7 ± 1.2	2.1 ± 3.7	1.1 ± 1.2	115 ± 62

**TABLE A6:** True lizard (Lacertidae) species and large‐scale densities at the main sites.

Main site	Species	Density/100 m^2^
1	*Podarcis muralis* (Laurenti, 1768)	< 0.1 lizards
2		0 lizards
3	Lizards indet.	< 0.1 lizards
4a	*Podarcis muralis*	< 0.1 lizards
4b	*Podarcis siculus* (Rafinesque‐Schmaltz, 1810)	2–3 lizards
5a		0 lizards
5b	*Podarcis siculus*	0.2–0.3 lizards
6	*Podarcis siculus*	< 0.1 lizards
7	*Lacerta trilineata* Bedriaga, 1886	< 0.1 lizards
8	*Lacerta trilineata*	0.1–0.2 lizards
9		0 lizards
10	*Lacerta trilineata*	0.2–0.3 lizards
11		0 lizards
12	*Algyroides nigropunctatus* (Duméril & Bibron, 1839), *Lacerta trilineata*	0.3–0.4 lizards
13	*Algyroides moreoticus* Bibron & Bory, 1833	0.5–0.6 lizards
14	*Podarcis erhardii* (Bedriaga, 1882), *Lacerta trilineata*	< 0.1 lizards
15	*Lacerta trilineata*	0.1–0.2 lizards
16	*Lacerta trilineata*	0.1–0.2 lizards
17	*Lacerta trilineata*	0.1–0.2 lizards
18	*Lacerta trilineata*	0.2–0.3 lizards
19		0 lizards
Mean		0.24 ± 0.54
